# Coastal Recreation in Southern New England: Results from a Regional Survey

**DOI:** 10.15351/2373-8456.1152

**Published:** 2022-08-01

**Authors:** Marisa J. Mazzotta, Nathaniel H. Merrill, Kate K. Mulvaney

**Affiliations:** 1 U.S. Environmental Protection Agency, Office of Research and Development, Atlantic Coastal Environmental Sciences Division, 27 Tarzwell Drive, Narragansett, Rhode Island, USA, 02882.

**Keywords:** Coastal Recreation, New England, Water Quality, Recreation Participation, Recreation Effort, Revealed Preference, Survey

## Abstract

This paper presents a summary of coastal recreation of New England residents from a survey conducted in the summer of 2018. The management of New England’s coasts benefits from understanding the value of coastal recreation and the factors influencing recreational behavior. To address this need, the survey collected the geographic location and trip details for both day and overnight visits to any type of location on the New England coast for a range of water recreation activities, providing a comprehensive view of coastal recreation in the region. This paper summarizes participation in various types of water recreation activities, including beachgoing, swimming, fishing, wildlife viewing, boating, and other coastal recreation activities. We quantify demand for coastal recreation using participation and effort models that disaggregate the dimensions of recreational behavior over space and census demographics. This provides insights on the scale and location of beneficiaries of this important human use of the natural environment. We found that 71% of people in the surveyed region participate in coastal recreation and engage in a wide range of coastal recreation activities at varied locations from open-ocean-facing coastal beaches to sheltered, estuarine ways to water. On average, people in the region take 37 trips to recreate on the coast of New England in a year, spending 167 hours per year visiting recreation sites and 66 hours traveling. This adds up to nearly 170.5 million trips from our sample region, 772.4 million hours of recreation time, and 304.6 million hours of travel time. Distance to the coast, demographics, and recreational activities affect how often people go and how much time they spend on coastal recreation.

## 1. INTRODUCTION

Despite the deep cultural importance of coastal recreation to New England residents, there is a lack of valid empirical economic and social studies quantifying these activities. Understanding the extent and value of recreational use supports decisions that affect coastal resources and allows for investigation of impacts from changes in water quality or other environmental conditions. Capturing the extent of recreation quantifies one aspect of the value of southern New England’s coastal ecosystem services, providing insights to managers about the importance of safeguarding marine resources and the benefits of doing so.

There are many studies in the environmental economics literature that quantify use and benefits, or willingness to pay (WTP), associated with water recreation. However, there are few recent studies that are relevant for the New England coast that include multiple recreational activities and types of access points. Many studies are freshwater based (e.g., Murray et al. 2001, Yeh et al. 2006, Egan 2009, Feather 1994, Melstrom and Jayasekera 2016). Other studies focus only on beaches (e.g., Bockstael et al. 1987, Parsons et al. 2009, Hilger and Hanemann 2008, Oh et al. 2008) or recreational fishing (Kaoru et al. 1995, Lipton and Hicks 2003, Bergstrom et al. 2004). Existing studies that address multiple types of recreation may not be relevant to New England policy questions because of dissimilar geographic study locations, or because they focus on aggregate benefits (e.g., Massey et al. 2017, Phaneuf 2002).

Most studies conducted in New England or nearby are dated (Bockstael et al. 1987, Kline and Swallow 1998, Opaluch et al. 1999). Opaluch et al. (1999) estimated per trip values for swimming, boating, fishing, and wildlife viewing on the East End of Long Island, and changes in WTP for swimming trips with changes in water quality. The estimates are based on a multiple site count-data model, which relied on assumptions that make welfare measures inconsistent with economic demand theory (Phaneuf and Smith 2005). Although they are dated, these estimates are still, to date, the most relevant values for coastal recreation days for different types of recreation, and that also address how values change with water quality changes for the northeastern United States.

While providing insightful case studies, the scopes for the more recent New England valuation and recreational use studies are limited geographically. Hwang (2018) estimated values per day for recreation in three Rhode Island salt ponds, and how values change with changes in water quality and congestion. Twichell et al. (2022) assessed differences in relative travel distances by census-block group demographics to public coastal amenities in Rhode Island. Lyon et al. (2018) modeled beach visitation on Cape Cod, Massachusetts as a function of weather, parking capacity, day of week, month, and beach closure history. Mulvaney et al. (2020) presented methods for estimating visitation to smaller access sites using targeted on-site counts. Merrill et al. (2020) and Furey et al. (2022) developed and applied methods for using human mobility data from cell phones to estimate visitation and visitors’ responses to beach closures for Cape Cod, Massachusetts. None of these studies provide recreational behavior estimates across the northeastern U.S.

Several studies at the national scale address participation and effort in coastal recreation (Leeworthy 2001, Leeworthy and Wiley 2001, Kosaka and Steinback 2018). While these studies provide a broad picture of New England’s coastal recreation, they do not address people’s choices of recreation site, WTP values, or effects of water quality on coastal recreation.

We conducted a survey of New England residents to capture an up-to-date and large-scale view of coastal recreation behavior. We collected information for participation in various types of water recreation activities, including beachgoing, swimming, fishing, wildlife viewing, boating, and other activities. In addition, we collected the geographic location and trip details for visits to the coast, allowing responses from trips to bathing beaches and other types of water access points for a range of water recreation activities, thus providing a more comprehensive view of coastal recreation in New England than currently exists. This paper presents a summary of New England coastal recreation, including models of participation—how many people participate in any type of coastal recreation, and effort—how often people recreate on the coast over the course of a year, as well as how far they travel and how much time they spend on coastal recreation. The results quantify multiple dimensions of behavior that reflect the demand for, and value of, coastal recreation, providing insights on the scale and location of beneficiaries of this important human use of the natural environment.

## 2. MATERIALS AND METHODS

### 2.1 Sample Description

**Figure 1.**
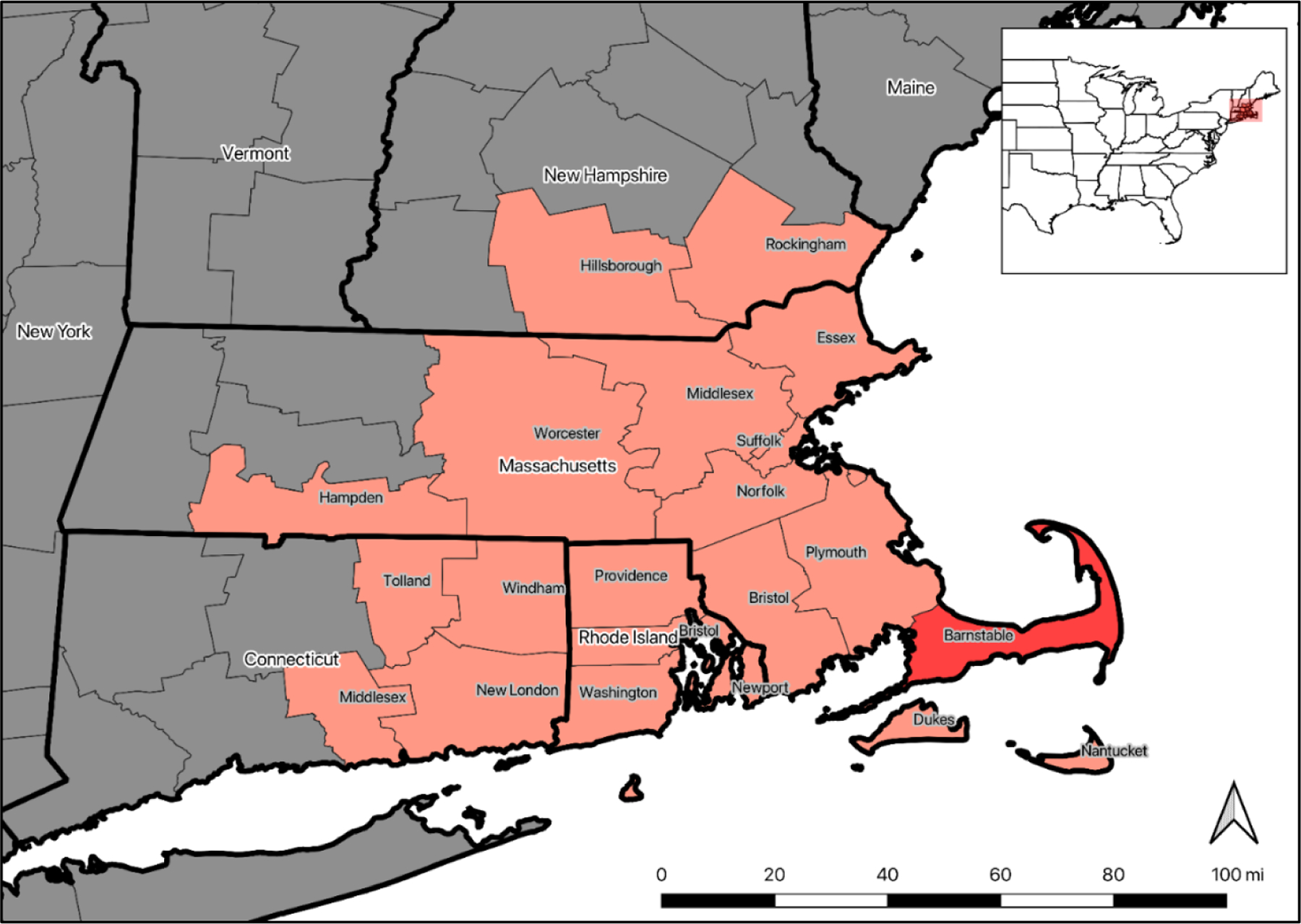
Map showing counties in Connecticut, Rhode Island, Massachusetts, and New Hampshire, USA that were included in the survey sample. Barnstable County, Massachusetts, was oversampled.

The geographic focus for the survey was Cape Cod, Massachusetts (Barnstable County), and New England residents within 100 miles of Cape Cod. We chose 100 miles based on typical driving distance to recreational sites (i.e., two hours or 100 miles) for single day or weekend trips. Parsons and Hauber (1998) found that the welfare relevant coefficients in a random utility model of water recreation are stable after the choice set is extended to around a two-hour travel distance. This was supported by our focus groups, and later confirmed in survey responses, where respondents stated that the farthest one-way distance they would travel on a single day is, on average, 71 miles and the longest one-way time is, on average, 1.7 hours. The sample region includes approximately 60% of all New England’s households including the urban centers of Boston, New Bedford, and Worcester, MA; New London, CT; and Providence, RI (U.S. Census Bureau 2021). Because the 100-mile radius from Cape Cod includes a large area of southern New England and many of its largest population centers, the results are more broadly applicable to residents of southern New England (Figure 1).

The survey was sent to a stratified random sample of 9,520 households in counties where more than 25% of the county’s geographic boundaries fall within 100 miles of Cape Cod, as measured from Bourne, Massachusetts, the farthest northwestern town on Cape Cod. The sample was stratified by geography, with Barnstable County sampled at a rate 3.06 times higher than the rest of the population in the study area. Households were selected randomly from the U.S. Postal Service Computerized Delivery Sequence File (DSF), the standard frame for address-based sampling (Iannacchione 2011, Link et al. 2008). The sample area includes two southeastern counties of New Hampshire, the eastern half of Massachusetts, all of Rhode Island, and the eastern half of Connecticut (Figure 1). We chose this geographic sample and oversample for Barnstable County for the purpose of travel cost and site-choice modeling as part of a larger research effort to value coastal recreation and water quality to support policy analysis in southern New England with a focus on Cape Cod (Barnstable County, Massachusetts).

Except where noted as survey sample statistics, results in this paper are corrected for demographics and sample weights to represent the population in our sampled area. We weighted responses with a base weight that adjusts for the probability of selecting a household in each county, combined with a calibration adjustment using demographic benchmarks and a raking method (Kolenikov 2016). The base weight corrects for oversampling of Barnstable County, and the calibration weight calibrates the results to be representative of household demographics in the study area. The final weights sum to the total number of households in the sampled study area (3,404,679).^1^

### 2.2 Survey Instrument and Sample Characteristics

**Figure 2.**
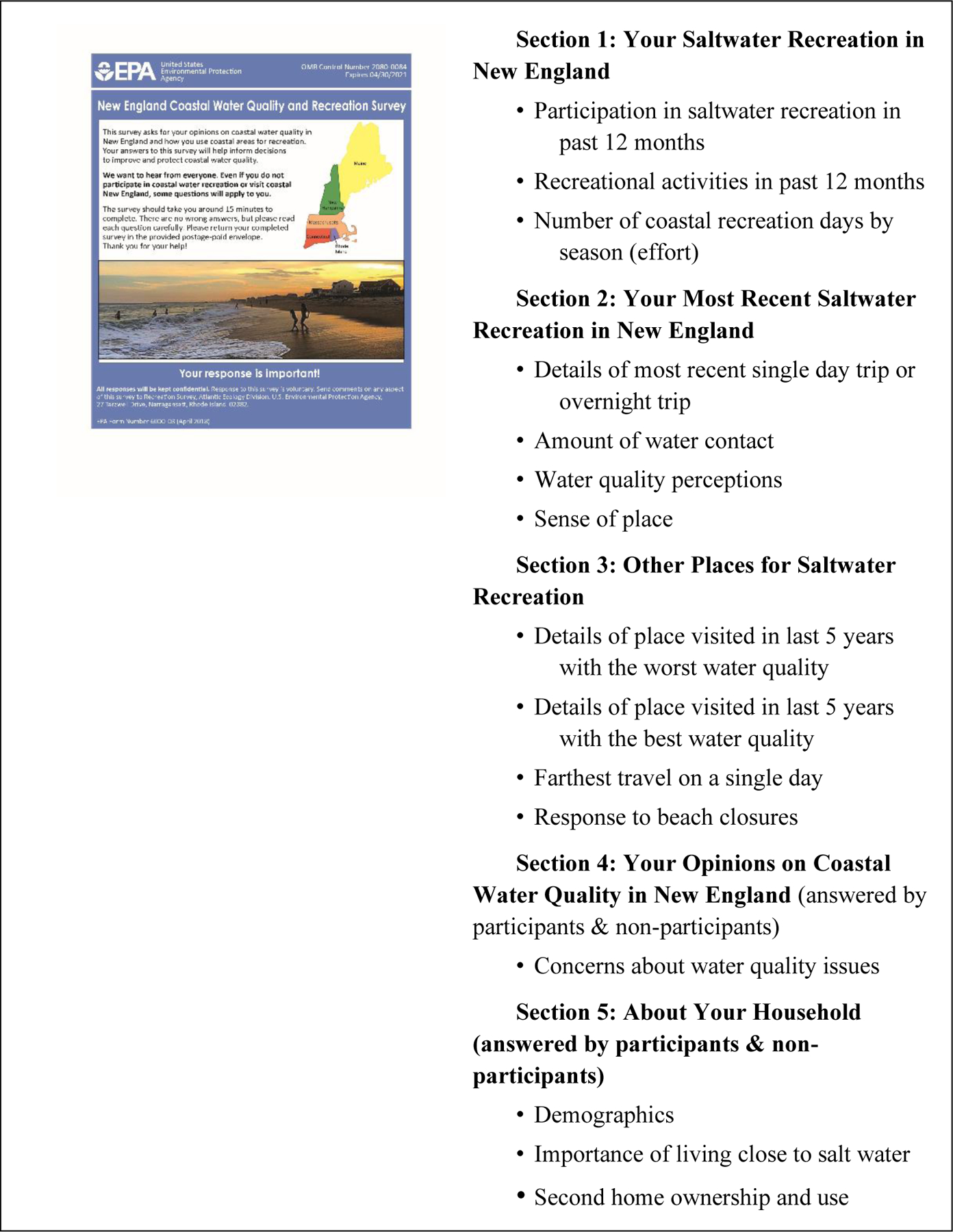
List of the 5 sections of the New England Coastal Recreation Survey questionnaire and their contents.

We developed the New England Coastal Recreation Survey questionnaire through a series of seven semi-structured focus groups (Desvousges and Smith 1988, Johnston et al. 1995) located within the study area – four in Rhode Island, two in Massachusetts, and one in Connecticut. The final survey included five sections that gathered: (1) participation and effort data for each respondent’s previous 12 months of saltwater recreation, (2) information on the last saltwater recreation trip taken, (3) water quality perceptions for other locations where the respondent has gone for saltwater recreation and other general coastal recreation questions, (4) the respondent’s opinions about a set of impacts of water quality issues in New England, and (5) demographic information (see Figure 2 for summary of survey content, Supplementary Materials SM1 for full questionnaire).

We used a mixed-mode approach to conduct the survey with a push-to-web initial invitation and paper survey follow up (Messer and Dillman 2011, Dillman et al. 2014). The first invitation letter was sent to an address and asked for one household member (over the age of 18) to log into the web survey and complete it online. We followed with a second reminder letter mailed one week after the initial letter and sent a final letter two weeks after that. The final letter included a paper version of the survey. We conducted an initial pilot survey in late May of 2018 to test the implementation logistics and survey questions and, after no needed changes to the questionnaire were identified, mailed the remainder of the survey sample from August to October, 2018. The mid-August to mid-September time frame for the bulk of the sample occurred towards the end of the New England summer recreation season, so likely captured most people who would have taken at least one recreational trip during the summer of 2018.

From a total of 9,520 surveys mailed, we received 1,437 responses to the pilot and main survey. After accounting for undeliverables, the response rate for all surveys was 15.74%. More than half of respondents (54.3%) answered through the web survey, while 45.7% answered on paper. Table 1 compares survey respondent demographics to census demographics for the study area population. Table A-1 compares demographics for those who answered the web and paper versions of the survey. The survey demographics overall are very close to the population of the study area, though survey respondents are older and more educated than the general population of the study area.

### 2.3 Statistical Models and Methods

In this paper, we present descriptive summary statistics, a logit model to predict participation in saltwater recreation, and a negative binomial model to predict effort (days per year spent by participants in saltwater recreation). The summary statistics use standard statistical measures, with weighting applied to correct for oversampling of Barnstable County and for demographic variations between our respondents and households in the survey sample area. We chose to separate the participation and effort models, rather than estimating a combined model, to maintain simplicity and interpretability, following similar analyses by Leeworthy and Wiley (2001). All statistical models were estimated using Stata/IC 16 (StataCorp 2019).

#### 2.3.1 Participation Model

We estimated a logit model to examine factors that affect the probability of participation in saltwater recreation in New England. The logit model is appropriate for discrete choices, such as the choice to participate or not (Greene 2000). It is specified as:

(1) $$P_{i}=\frac{1}{1+e^{-X_{i}\beta_{i}}}$$

where

P_i_ = the probability that a person participated in activity i in the previous 12 months

X_i_ = a vector of characteristics of the household

β_i_ = a vector of estimated parameters

#### 2.3.2 Effort Model

We applied a zero-truncated negative binomial model to estimate factors that affect the number of days spent engaging in saltwater recreation in New England, a model appropriate for the count type of data reported in the survey. Because the variance and mean of the outcome variable are not equal, we applied the negative binomial model rather than a Poisson model. The zero-truncated model accounts for data with no zeros, as everyone who participated spent at least one day engaging in recreation (Grogger and Carson 1991).

## 3. RESULTS

### 3.1 Participation

When weighted, we estimate that 71.2% of the population of our survey sample area, over 2.4 million people, participated in saltwater recreation in New England in the 12 months preceding our survey. By state, the weighted participation rates are 75.7% for New Hampshire, 74.9% for Rhode Island, 70.5% for Connecticut, and 70.1% for Massachusetts. Participation declines with distance from coast, with the highest participation among people who live within 2 miles of the coast (Table 2). Table A-2 provides statistics on average distance to the coast by state. Tables A-4 and A-5 break down participation rates and days spent in coastal recreation by those who answered the web survey or paper survey.

**Figure 3.**
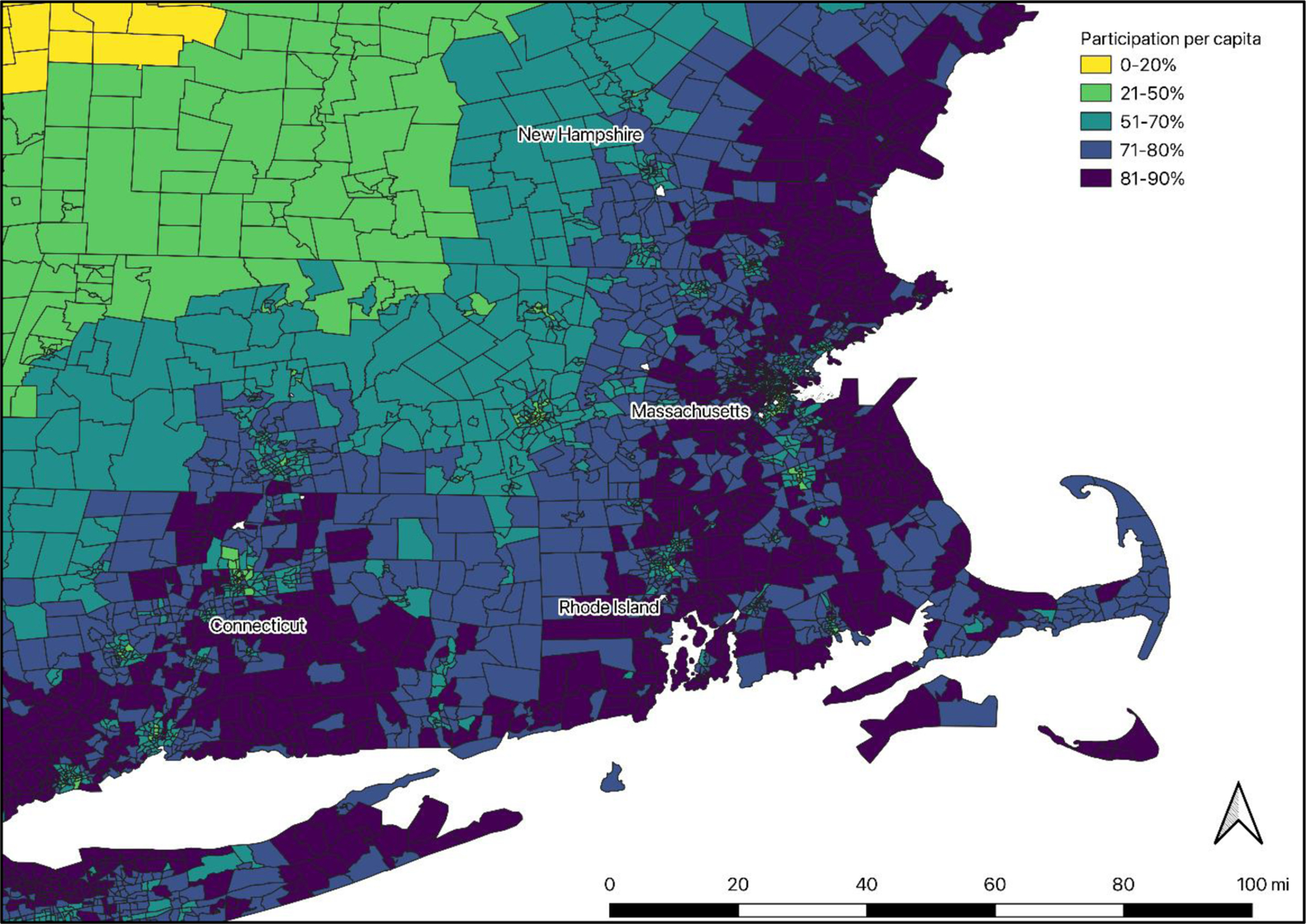
Predicted per capita participation in coastal recreation in New England by census tract. The map shows participation rates for each census tract calculated by applying the logit participation model (Table A-7) using census data and calculated straight-line distances to the coast.

Using a logit model, we estimated how demographic variables affect participation in saltwater recreation in New England in order to predict how participation might change with changes in demographics. The model was estimated using weights, as described above, and model outputs are included in the Appendix (Table A-7). Table A-6 provides summary statistics for dependent variables in the participation and effort models. We found that proximity to the coast, age, race, education, and income had significant impacts on participation. There is a decline in the probability of participation for people who live farther from the coast (using straight-line distance to the coast from the census block where the survey was mailed). Participation increases with age up to around age 60, and then decreases. Those who identify as non-white (including mixed non-white races) are less likely to participate than those who identify as white. People with four-year college degrees or graduate degrees and those with household income of $100,000 or higher are more likely to participate than those with lower education levels or incomes. In initial modeling tests, we included individual states in the model as covariates, but found no significant differences in participation across states. We mapped predicted participation by census tract by applying the model coefficients to census demographic data and calculated straight-line distances to the coast (Figure 3; U.S. Census 2021).

The reported coefficients in the logit model, presented in the Appendix (Table A-7), are the log of the odds of participating and thus are not straightforward to interpret. To provide more readily interpretable results, we present marginal effects from the model in Table 3. These are interpreted as the effect on the conditional mean value of y—whether someone participates—with a change in a regressor, holding all other variables at the mean sample value (equivalent to the slope coefficients in an OLS model). The statistically significant results indicate that a person is about 0.5% less likely to participate in coastal recreation for every mile farther from the coast that they live and about 0.5% less likely to participate for every year increase in age.

People with a college or graduate degree are about 15% more likely to participate, and those with household income of $100,000 or higher are 9% more likely to participate. People who identify as non-white are 42% less likely to participate than those who identify as white only or mixed race including white. This characterization of participation in coastal recreation with respect to demographics is largely consistent with past work on coastal recreation and may reflect disproportionate allocations of resources across demographic groups in terms of shoreline access and disposable income and time (Twichell et al. 2022, Montgomery and Chakraborty 2015).

### 3.2 Activities

Table 4 shows the weighted estimates of participation in different saltwater recreation activities for our study area – the percent of people 18 years old or older estimated to participate in each activity, and the number of people this represents (the total number of people age 18 and over in our study area is 6,506,166; U.S. Census 2021). The most popular activities are activities on the shore, followed by swimming, wading, wildlife viewing, kayaking/canoeing/rowing, fishing, and motorboating. Participation rates by activity for various demographic groups are shown in the Appendix (Table A-8).

### 3.3 Effort

We asked those who had participated in the last 12 months to estimate how many days they spent doing saltwater recreation in New England during each season of the last 12 months, using an open-ended format. Table 5 shows the weighted estimates of days per year for the entire survey sample area, and the number of people age 18 and over included in each category. As expected, most days occurred during the warmer months. When projected to our entire surveyed area, we estimate that people in our sample region spent over 170 million days engaged in coastal recreation in New England in the 12 months prior to our survey (2017–18). Twenty-five percent of respondents who participated in coastal recreation spent 6 or fewer days per year, and 25% spent 65 or more days per year; 50% spent between 6 and 65 days per year. The number of days spent decreases with distance from the coast (Table 6).

We estimated a truncated negative binomial regression to predict total number of coastal recreation days per year for participants. The model results, which explain the log of total visits, are presented in the Appendix (Table A-9), and we present the marginal results here. The first model presented includes the same demographic variables included in the participation model. Variables that are significant in the effort model include distance to the coast, age, and household income.

**Figure 4.**
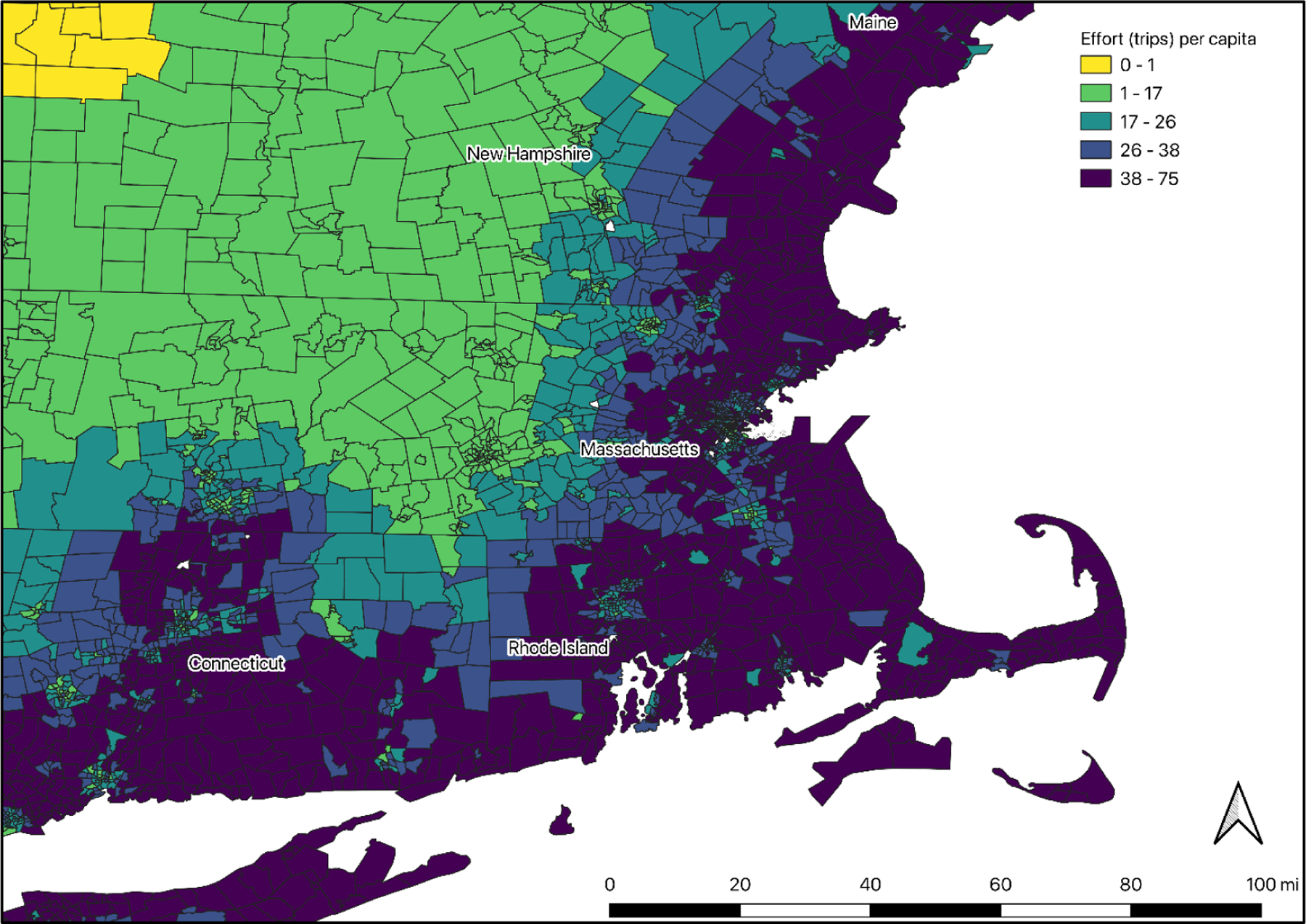
Predicted per capita days spent per year for those that participate in coastal recreation in New England by census tract. The map shows per capita days per year for each census tract calculated by applying the effort model (Table A-9) using census data and calculated straight-line distance to the coast.

The marginal results show that, for every additional mile lived from the coast, people spend 1.4 fewer days per year on saltwater recreation. For every additional year of age, people spend about 0.6 more days per year. Those who identify as non-white spend 15.4 fewer days per year, and those with household incomes of $100,000 or higher spend 17.5 more days per year. Figure 4 maps the results of this model to census tracts, to illustrate variations in days spent engaging in saltwater recreation (U.S. Census 2021).

We estimated a second model that includes variables for different categories of activities and for whether the respondent owns a second home at the coast (Tables A-10 and A-11). This model indicates that people who participate in birding and other wildlife viewing; fishing, shellfishing and hunting; and board sports spend significantly more days per year engaging in coastal recreation. Those who are wildlife viewers spend the most days on coastal recreation, followed by those who fish or hunt, those who engage in board sports, water immersion activities, non-motorized boating, and motorized boating.^2^ Table A-12 shows results of an OLS regression that examines factors that may be related to the amount of time spent on day trips.

### 3.4 Locations of Last Coastal Recreation Trip

**Figure 5.**
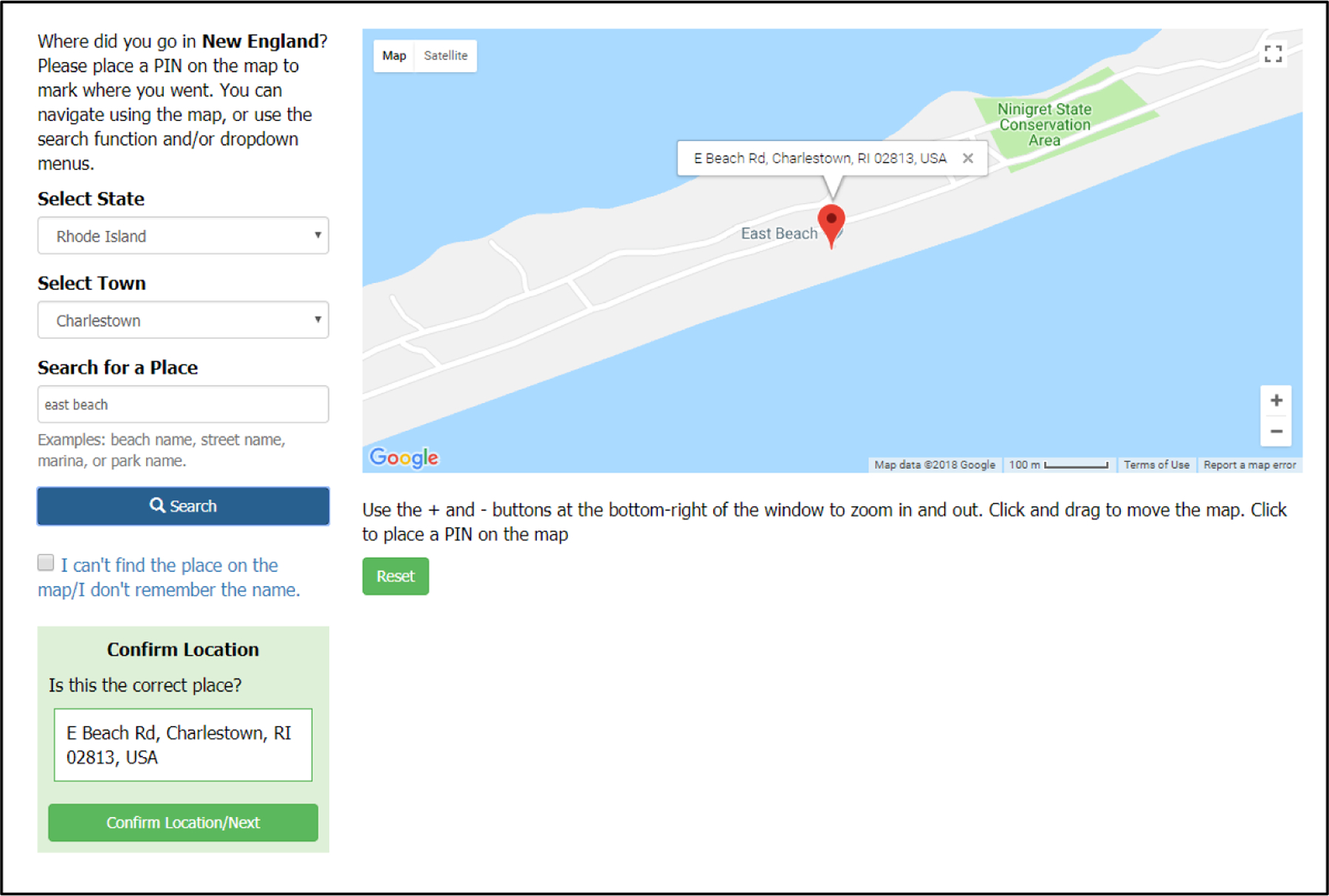
Screenshot of web survey mapping page where respondents could enter the location of their last trip using an interactive map.

A novel aspect of our survey was its ability to capture responses for trips to any coastal access point in New England, as opposed to the often-used practice of providing a list of recreational locations from which respondents select. The online survey included interactive mapping functionality that allowed for scrolling to a location and dropping a pin or using a search box to locate the place where the person had last recreated along the New England coast (Figure 5). Asking for the last trip allowed us to get a sample of trips across a variety of types of access points and activities, as opposed to asking for specific types of trips or locations. This is especially important for evaluating policies that affect the many smaller coastal access points that frequently are located in estuaries and can serve large numbers of users (Mulvaney et al. 2020). They may be located in urbanized areas, where residents may find it difficult to travel to and access open-water coasts. Many water quality issues and other environmental issues in coastal New England occur in estuaries and more contained waters and not at major, open-water beaches. The paper survey asked people to write in the location visited, using state, city or town, and name of the specific location. We were able to convert 85% of these “write-in” locations to specific locations (latitude/longitude coordinates) compatible with the online mapped locations using the combined text of the write-in information as input to Google Map’s Application Programming Interface (API) geocoding search (Google 2020).

**Figure 6.**
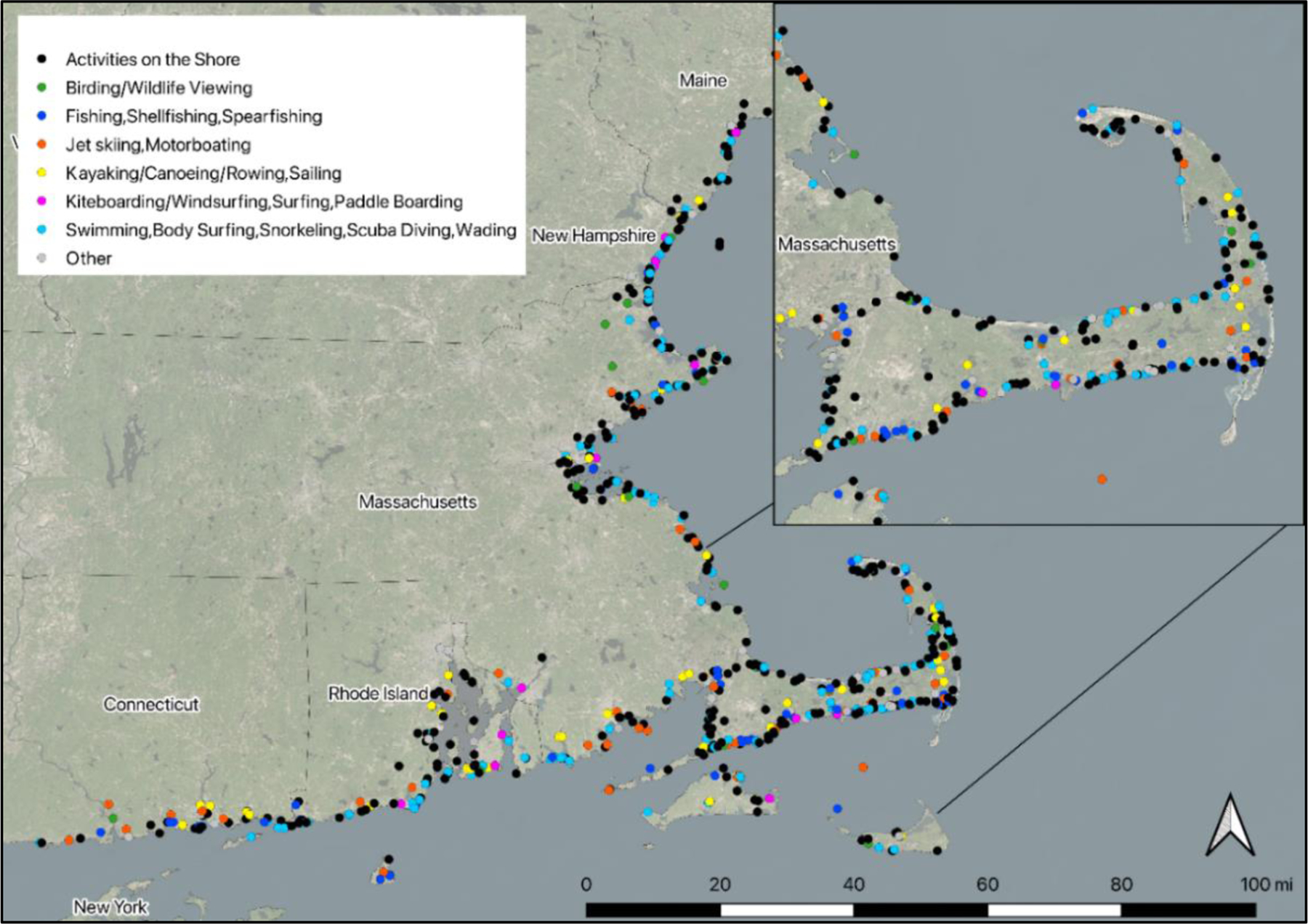
Map showing locations visited by survey respondents for coastal recreation, with their primary activities indicated by the color of the dots. Note that the dots may overlap. Figures A1–A8 in the Appendix show locations visited by activity group.

**Figure A-1.**
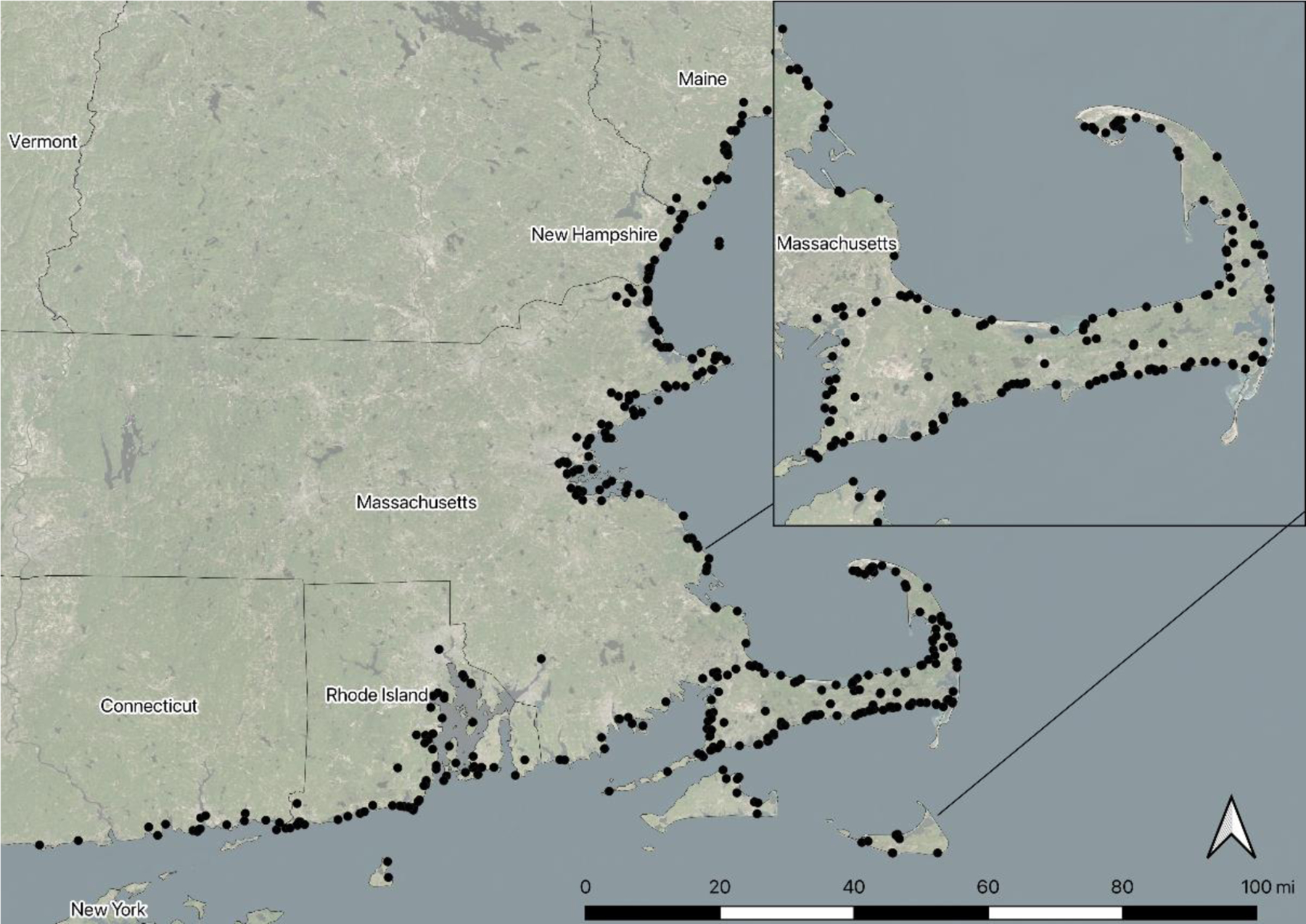
Activities on the shore.

**Figure A-2.**
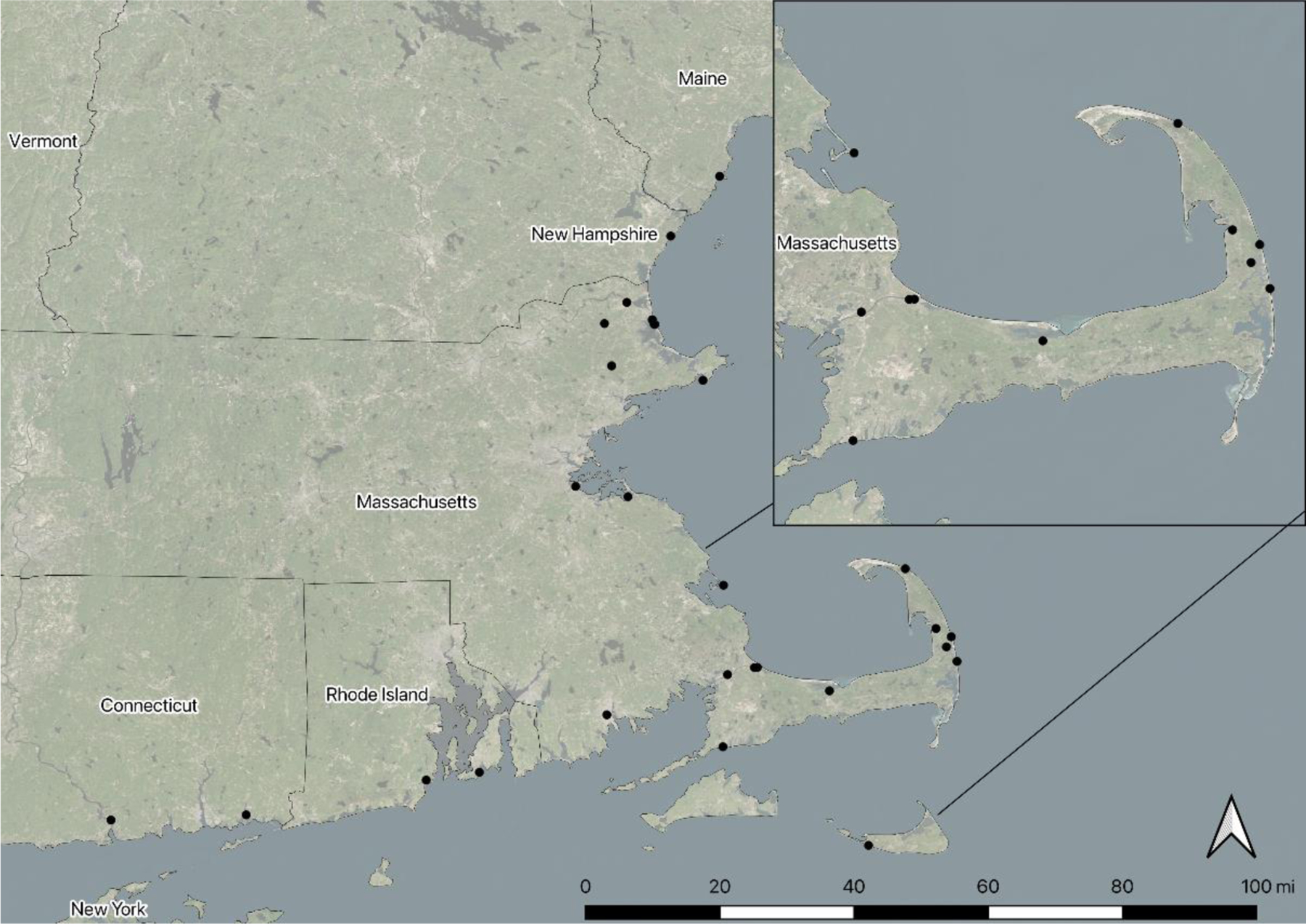
Birding and wildlife viewing.

**Figure A-3.**
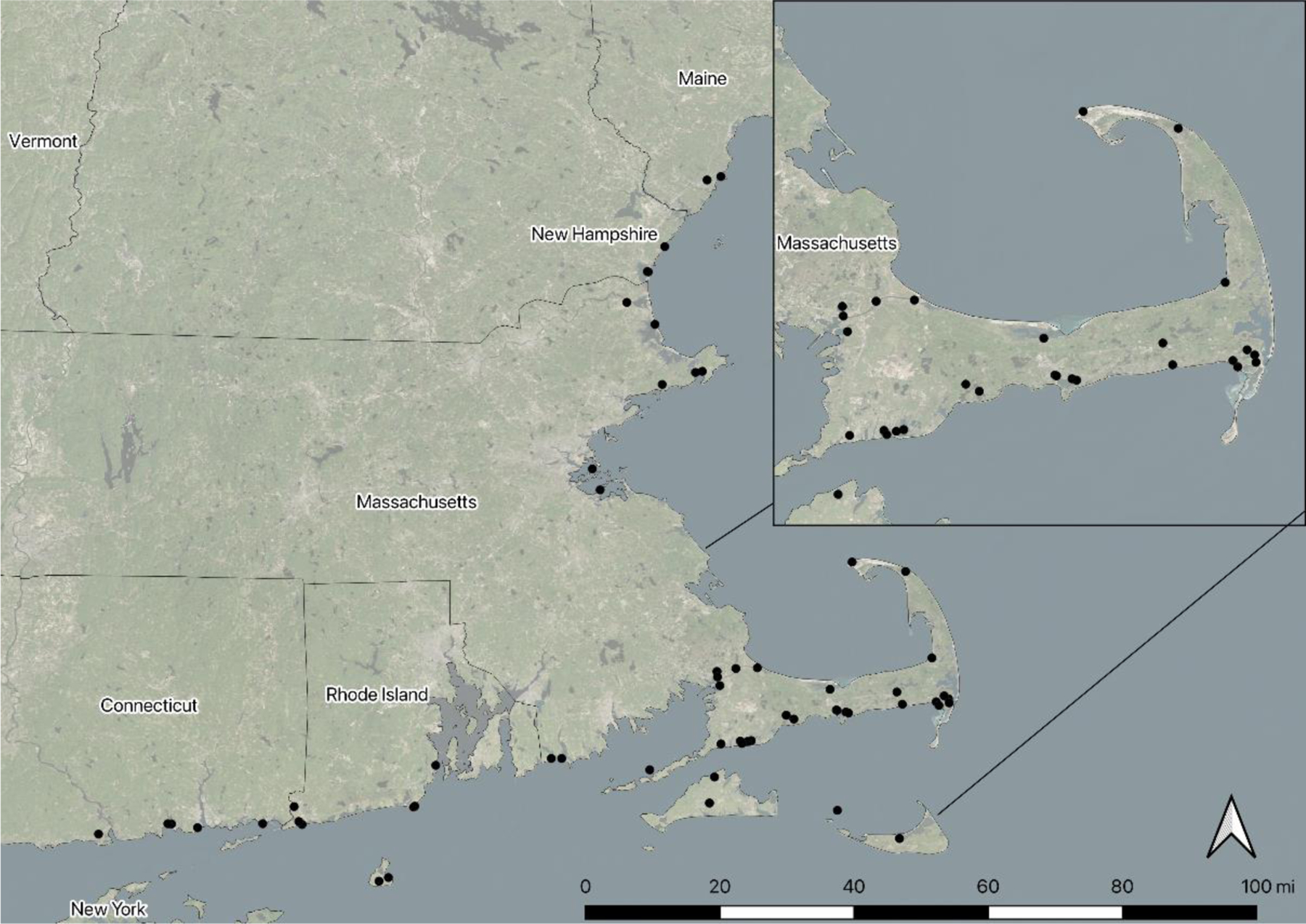
Fishing and shellfishing

**Figure A-4.**
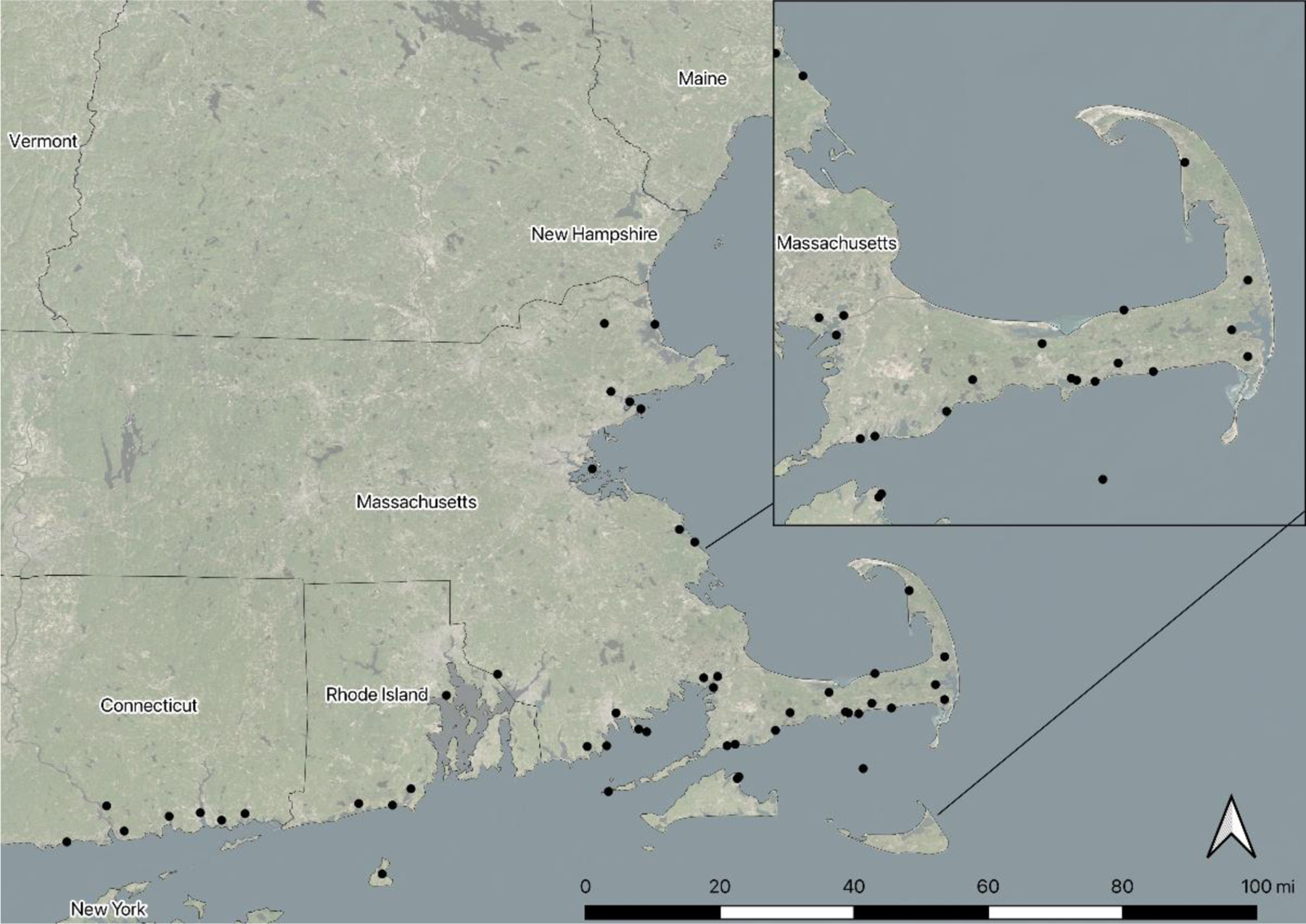
Motor boating and jet skiing.

**Figure A-5.**
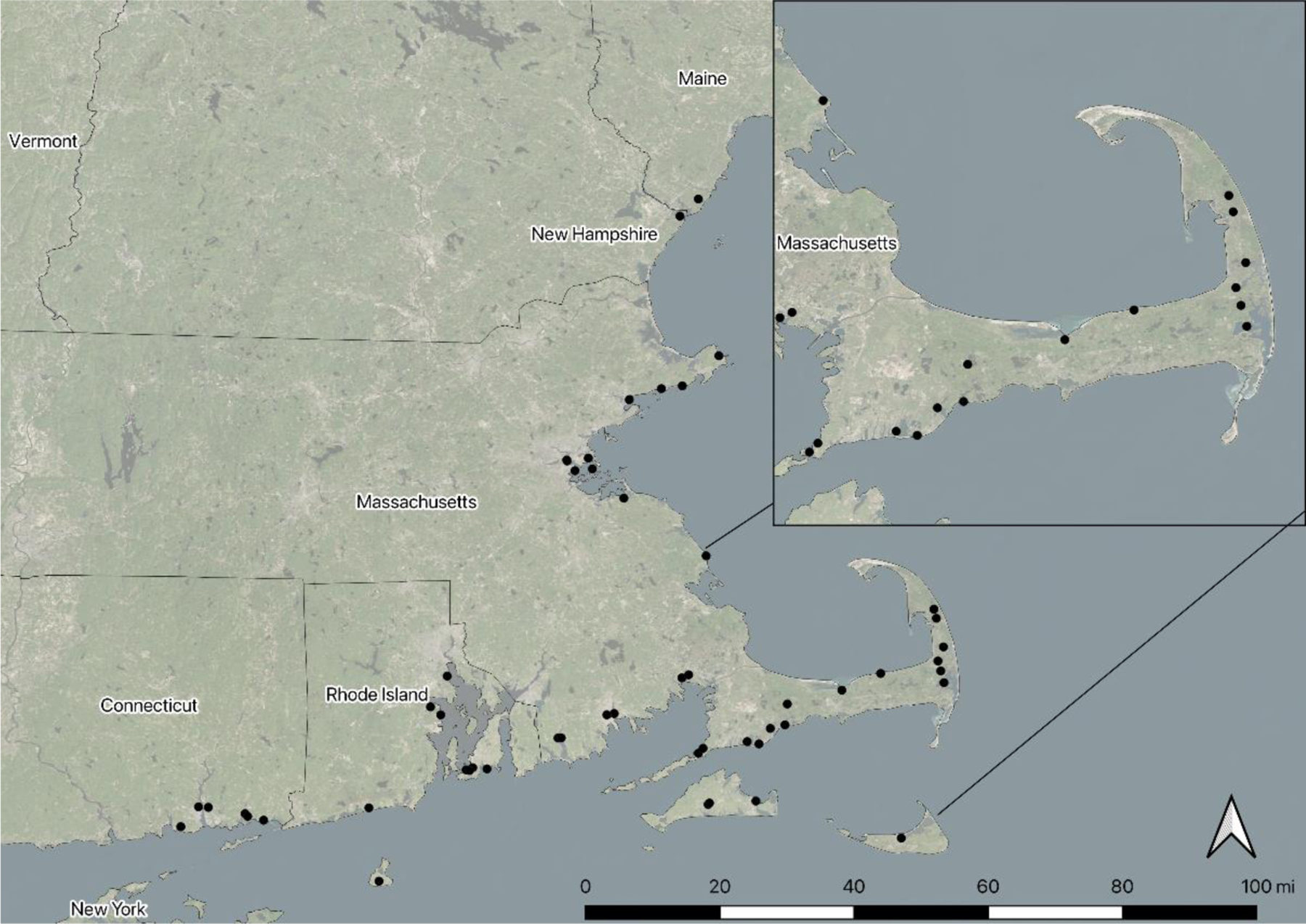
Kayaking, canoeing, rowing, sailing.

**Figure A-6.**
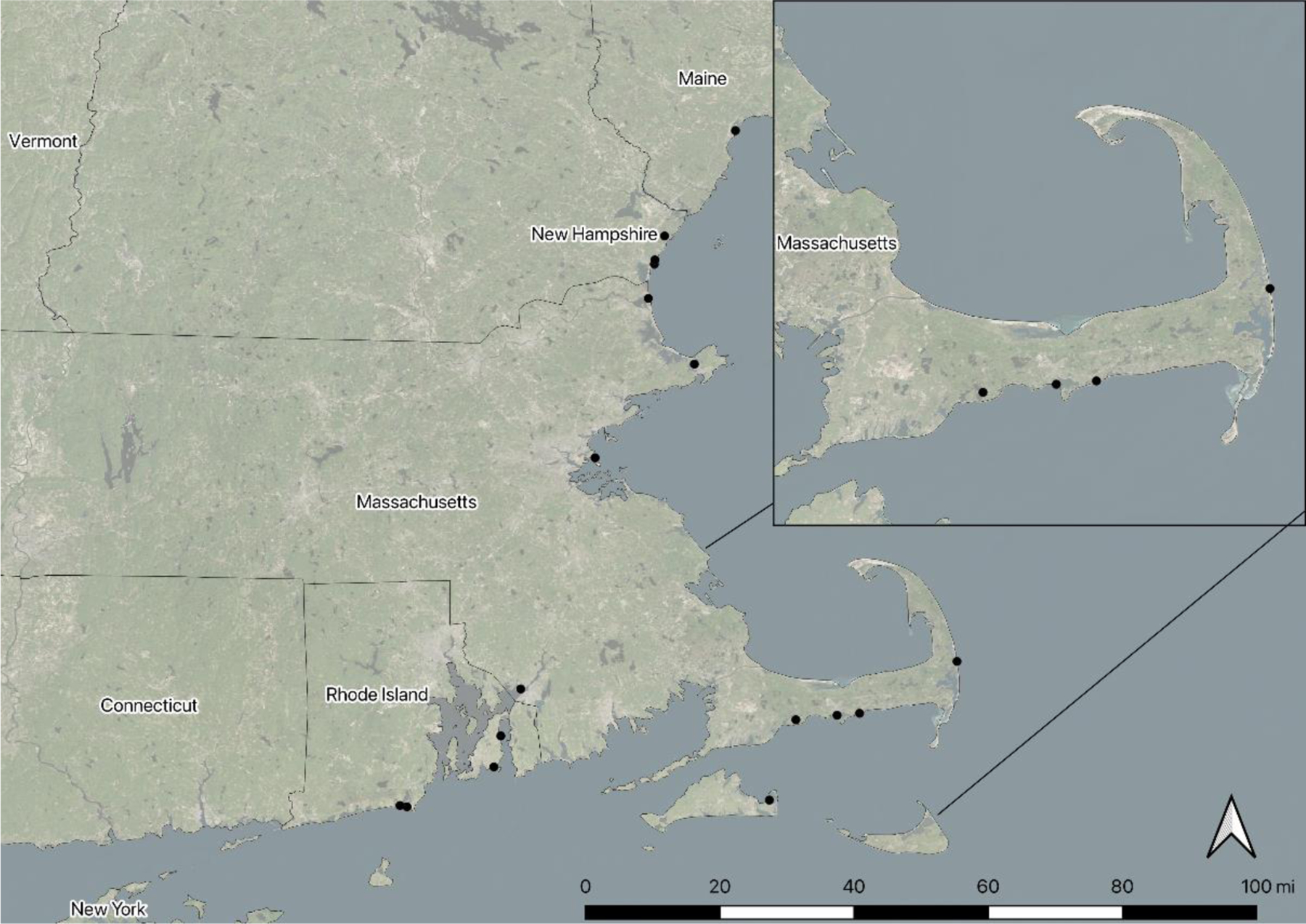
Board sports.

**Figure A-7.**
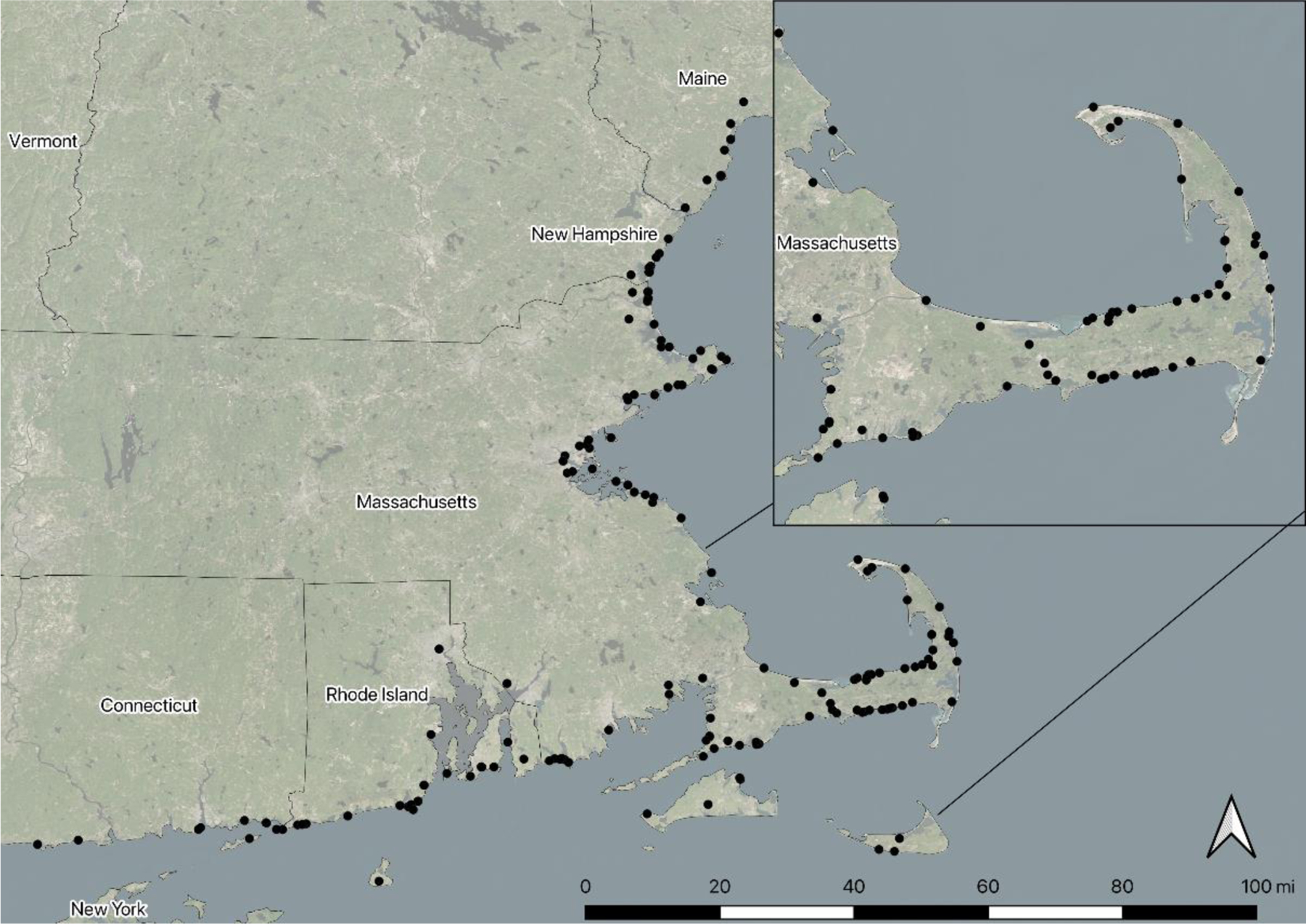
Swimming, body surfing, snorkeling, scuba diving, wading.

**Figure A-8.**
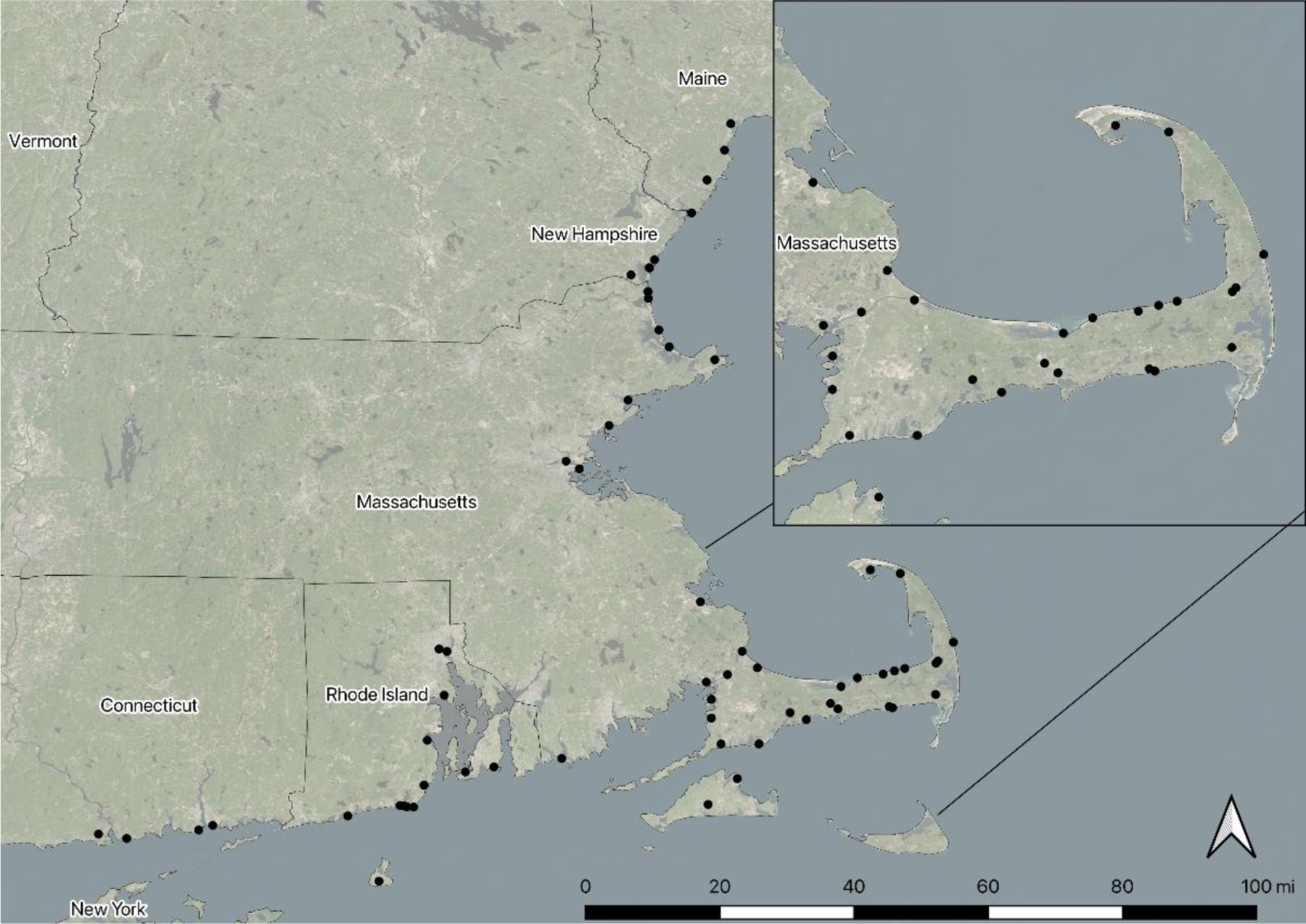
Other coastal recreation activities.

Figure 6 is a map of the locations visited and primary activities for respondents’ most recent trip, including the last recreation day during a multiple-day trip. Figures A1–A8 break this down by activity groups. Table A-3 presents the percent of trips to each state by the state of origin. Using secondary data sources, we linked coastal and shoreline attributes to the locations people visited for their reported trip (see Supplementary Materials SM2 for details).^3^ Seventy percent of trips were to locations that include a designated swimming beach (defined as beaches included in EPA’s BEACON database; U.S. EPA 2021). Forty-two percent are classified as “sheltered” locations, as opposed to locations exposed to wave and tidal energy (NOAA 2019). Sixty-six percent of locations are classified as having at least one water quality impairment on the state’s 303(d) listed waters under the Clean Water Act.^4^ A portion of a water body may be impaired for various reasons, including fecal coliform, dissolved oxygen, or fish bioassessments, for example.

**Figure 7.**
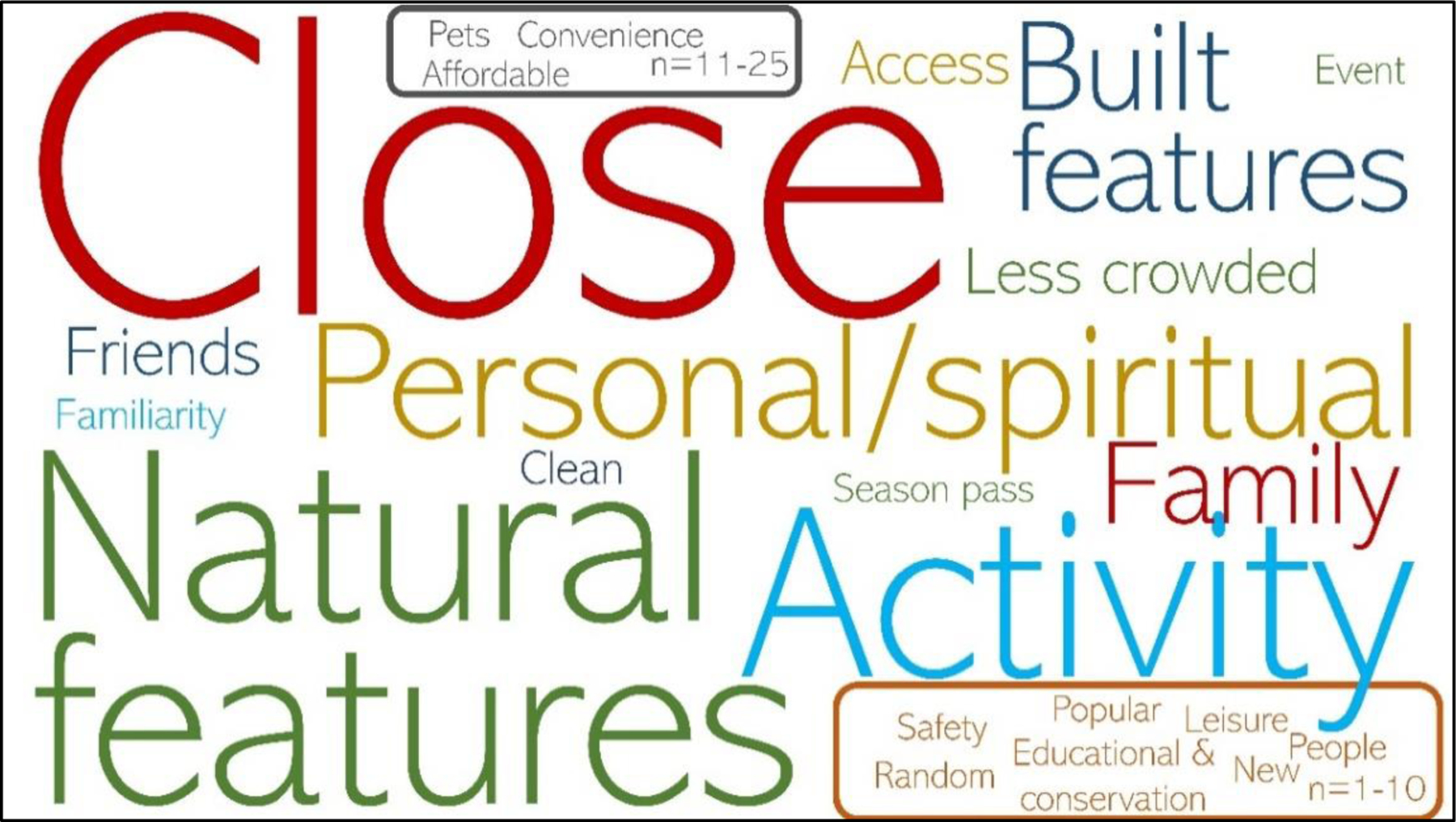
Word cloud of coded responses to “Why did you choose that location?” Font size is proportional to each code’s number of responses, although any code with 25 or fewer responses is presented using the same font size for legibility. The coded responses that were identified by 1 to 10 participants are encircled in the bottom right corner and colored orange. The coded responses that were identified by 11–25 participants are encircled in the center top and colored gray. All other coloration is random.

When asked in an open-ended question why they chose a particular location, respondents noted a number of different reasons, including reasons related to site characteristics, their companions, and specific activities (Figure 7). The 826 people who responded to the question provided a total of 1,228 reasons (some respondents provided multiple reasons), which we coded into 23 categories (see Supplementary Materials SM2 for details on the codes). Proximity (coded as “close”) was the most commonly identified reason (n=289), followed by natural features of the site (n=165), suitability for the activity they were interested in (n=164), and spiritual and personal reasons (n=111). “Clean” was mentioned by 32 people. These responses highlight the importance of supplementing travel cost methods with studies that investigate the various social values of coastal visitation. Although affordability is related to distance, only 12 respondents mentioned cost or affordability explicitly.

### 3.5 Distance Traveled and Trip Duration

Although respondents identified proximity as important for site choice, the respondents in our sample spent considerable time and effort getting to their coastal recreation location, as well as time spent on site. Time spent and distance traveled represent monetary and non-monetary costs to people. Quantifying how much of these limited resources people allocate to coastal recreation reflects the scale of the value they hold for it.

Table 8 shows the one-way distance traveled in miles, and time traveled in minutes for day trips^5^. The table shows both the distance and time reported by people on the survey and the distance and time from the respondent’s home to the chosen coastal recreation location, which we calculated via road networks using a local build of an Open Source Routing Machine (Luxen and Vetter 2011). People’s unweighted mean reported distance and time are slightly less than the calculated mean distance and time, but are remarkably close.

Respondents reported that the farthest one-way distance they would travel for a day trip was, on average, 71 miles with a median of 60 miles. They reported that the maximum one-way time they would spend traveling for a day trip was 101 minutes (1.7 hr.), on average, with a median of 90 minutes (1.5 hr.).

Table 9 summarizes overall participation, time spent, and miles traveled in the study region (which includes approximately 60% of the New England population). On average, people in the sample area spent 4.5 hours on site during a day trip, and 1.8 hours of round trip travel time. For the nearly 170.5 million trips per year taken by those 18 and over (N = 4,629,453), this equates to over 772 million total hours over the year engaging in coastal recreation and over 12.5 billion miles and 304.5 million hours in transit. NOAA’s 2012 survey of coastal recreation found that the average daily trip expenditure for New England coastal recreation was close to $70 (2021$; Kosaka and Steinback 2018). For our total estimated trips, this sums to over $11.8 billion in annual expenditures.

## 4. DISCUSSION AND CONCLUSIONS

Coastal recreation plays a significant role in southern New England’s culture and economy. We estimate that people age 18 and over spend close to 170.5 million days per year in coastal recreation along the coasts of New England, with more than $11.8 billion in direct expenditures. This does not include consumer surplus for recreational trips, which is the subject of future work stemming from this survey collection. The wide range of activities and types of coastal areas visited reported by the survey respondents demonstrates that coastal recreation encompasses much more than just beachgoing. The scale and scope of coastal recreation highlights the importance of maintaining the availability and quality of coastal resources.

To put our survey results in perspective, the closest comparable information comes from NOAA’s 2012 National Ocean Expenditure Survey (Kosaka and Steinback 2018), based on a web-based research survey panel as opposed to our mailing address-based sample. They estimated that 4.5 million New England residents participated in coastal recreation in New England in 2012, while we estimate that 4.6 million residents of our surveyed area participated in 2017–18. They found that New England residents who participated in coastal recreation in 2012 did coastal activities an average of 25 days per year per person, compared to 37 in our survey. While the participation questions were similar on our survey, the NOAA survey asked about effort over shorter time-periods (one month) in waves, potentially improving the accuracy of recall as compared to our 12-month recall period. Also, as our survey results showed, participation and number of days declines with distance from the coast, and our sample included people within 52 miles of the coast (representing around 60% of the New England population) whereas their sample included all of New England.

Sea-level rise, erosion, and coastal development all threaten the availability and quality of recreational resources (Bowker and Askew 2013). Given the high levels of participation and effort shown in this survey, mitigating these effects may bring large social benefits. Often, improving recreational opportunities and experiences are cited as benefits to restoration, pollution cleanups and improving access. However, there is little quantifiable information on the scale and importance of those recreational activities. The findings from this survey provide a snapshot of how people in southern New England use coastal resources and highlight the benefits of maintaining and improving the quality of the coastal environment.

Survey methods, such as the ones we used, suffer from sampling issues due to the nature of the instrument. Mail surveys, or in our case a mail with push-to-web survey, have had falling response rates over time (Stedman et al. 2019). As response rates fall, the representativeness of the sample to the general population becomes more tenuous. We controlled for this by following standard survey methods described above, but there is a need for complementary methods, such as intercept surveys and targeted focus groups to understand the perspectives of people not easily sampled in a traditional survey. Similarly, while we can collect specific and individual-level detail by surveying people, quantifying overall visitation levels to particular places requires a different kind of data collection. Onsite counts and new methods of capturing human behavior, such as using cellular device locational datasets, provide complementary information on important dimensions such as understanding overall visitation levels to specific places (Merrill et al. 2020; Mulvaney et al. 2020; Lyon et al. 2018).

Large, one-off mail surveys provide a detailed snapshot in time, but tell us little about changes in behavior or trends around changes in environmental quality. For that, repeated or ongoing surveys could provide more context across time. For example, this survey was conducted before the COVID-19 pandemic, where behavior and people’s time management clearly shifted. Understanding what changed and how durable these changes are, would take additional repeated work. Similarly, understanding the behavioral impact of improvements or degradations in environmental quality in the coastal zone may require additional data collections. While there was no prior baseline data covering the entire coast of the study area, for any activity, our survey can provide a baseline for future studies.

We designed the effort and participation models as a function of demographics to understand the makeup of coastal users as well as to project recreational demand from U.S. Census information. There is more to learn about how different types of people use coastal resources and about the places and their environmental quality that support those activities. Transportation and time represent considerable costs to entry, even before any potential parking and entry fees. These costs show the value people place on these activities, but also can represent barriers to entry. If people have to travel farther to find quality coastal recreational opportunities, a burden is imposed that may be addressed with targeted restoration of access and quality in proximity to population centers (Twichell et al. 2022). As noted earlier, participation in coastal recreation is not equally distributed throughout the population. In particular, respondents who identified as people of color were less likely to participate than white respondents. These differences in use may reflect differences in recreational preferences, but also inequity in access and other systemic barriers.

This paper has presented summary results from a survey of coastal recreation in southern New England, providing a needed baseline for quantifying coastal recreation and its benefits in the region, and highlighting the importance and value of coastal resources. The data can be used in conjunction with other types of data collections to get a more complete picture of this important cultural resource. Future work will use the data to estimate monetary benefits of coastal recreation and values for improvements in water quality.

## Supplementary Material

Supplement1

Supplement2

## Figures and Tables

**Table 1.** Survey Sample Demographics Compared to Study Area Population Demographics

Demographics	Survey Respondents	Study Area Population
Male	47.5%	49%
Female	52.5%	51%
Median Age	59	48
White	86%	82%
High School Education	33%	24%
Bachelor’s Degree	32%	27%
Graduate Degree	34%	17%
Employed	58%	64%
Retired, student, unemployed	42%	34%
Household Income		
$0–99.9k	56%	59%
$100–199.9k	22%	28%
$200k and over	13%	12%

Note: Study area demographics are from US Census 2014–2019 American Community Survey 5-year estimates for the sampled counties (U.S. Census, 2021). Census data were weighted by county using the survey base weights.

**Table 2.** Participation Rates by Distance from Residence to Coast

Distance from Residence to Coast	Mean	Linearized Standard Error	95% Confidence Interval	
up to 0.5 mile	76.5%	2.7%	71.1%	81.8%
from 0.5 mile to 2 miles	76.4%	2.7%	71.1%	81.7%
from 2 miles to 10 miles	69.2%	2.8%	63.7%	74.8%
from 10 miles to 20 miles	64.8%	3.7%	57.4%	72.1%
farther than 20 miles*	63.5%	4.2%	55.1%	71.8%

* The maximum distance from the coast for respondents was 52.2 miles.

**Table 3.** Marginal Effects of Respondent Characteristics on Participation

Variable	Marginal Effect	Standard Error	z	P>|z|	95% Confidence Interval	
Distance from residence to coast (mi.)	**−0.005**	0.001	−3.52	0.000	−0.008	−0.002
Household size	0.017	0.015	1.10	0.270	−0.013	0.047
Age	**−0.005**	0.001	−4.80	0.000	−0.007	−0.003
Female	−0.004	0.030	−0.12	0.906	−0.063	0.056
Non-White	**−0.425**	0.071	−6.02	0.000	−0.563	−0.287
4-Year college or graduate degree	**0.152**	0.035	4.370	0.000	0.084	0.221
Household income $100,000 or higher	**0.090**	0.033	2.77	0.006	0.026	0.154

Note: Marginal effects are calculated with other variables at their mean levels (see Table A-6 for summary statistics). Marginal effects for binary (dummy variables) are for the discrete change from the base level.

**Table 4.** Estimated Percent and Number of People in the Study Area Who Participate in Various Coastal Recreation Activities

Activity	% of People Who Participate	Estimated # of People Who Participate*
Any activity	71.2%	4,629,453
Activities on the shore	62.0%	4,033,403
Swimming/Body surfing	42.5%	2,763,546
Wading	34.8%	2,261,090
Birding/Wildlife viewing	21.6%	1,405,426
Kayaking/Canoeing/Rowing	15.4%	1,000,879
Fishing	14.3%	930,434
Motorboating	11.7%	760,083
Surfing/Boogie boarding	7.6%	496,035
Sailing	7.0%	457,010
Paddleboarding	5.0%	327,254
Shellfishing	4.5%	294,084
Snorkeling	2.7%	176,706
Tubing/Waterskiing	2.4%	156,945
Jet skiing	1.6%	102,132
Scuba diving	1.3%	82,720
Skimboarding	1.2%	79,891
Other	1.0%	61,890
Hunting	0.7%	44,478
Kiteboarding/Windsurfing	0.5%	29,945
Spearfishing	0.1%	9,047
Did not participate	28.8%	1,876,713

* The totals include people age 18 and older.

**Table 5.** Estimated Days of Coastal Recreation per Season and Year for Survey Sample Area

Season	Mean Days Spent*	Number of Participants**	Estimated Total Days***
Spring (March, April, May)	8.95	3,401,893	30,449,820
Summer (June, July, August)	18.83	4,252,072	80,066,906
Fall (September, October, November)	10.16	3,537,166	35,946,516
Winter (December, January, February)	3.29	2,981,586	9,822,978
12 month total	36.83	4,629,453	170,484,872

* To eliminate outliers, total days were truncated to remove values >95^th^ percentile. Means of seasonal responses include zeros. When calculating the 12 month mean, seasonal estimates for each person were added to get the 12 month total per respondent. Zeros were not included when calculating the 12 month mean, since by definition a participant needed to spend at least one day recreating in the calendar year.

** The number of participants was calculated by using the survey demographic and sampling weights to estimate the number of households in the sample area who participated in each season and in the last 12 months. This was multiplied by 1.91, the number of people age 18 and older per household (from the U.S. Census).

*** Total days for each season and for 12 months were calculated by multiplying mean days by number of participants.

**Table 6.** Days Spent Engaging in Coastal Recreation per Year by Distance from Residence to the Coast

Distance from Residence to Coast	Mean	Linearized Std. Error	95% Confidence Interval	
Up to 0.5 mile	61.5	4.2	53.2	69.8
From 0.5 mile to 2 miles	37.9	3.7	30.7	45.1
From 2 miles to 10 miles	27.4	3.2	21.2	33.6
From 10 miles to 20 miles	20.9	3.7	13.7	28.0
Farther than 20 miles*	19.3	3.3	12.9	25.8

* The maximum distance from the coast for respondents was 52.2 miles.

**Table 7.** Marginal Effects of Respondent Characteristics on Days Spent Recreating

Variable	Marginal Effect	Standard Error	z	P>|z|	95% Confidence Interval	
Distance from residence to coast (mi.)	**−1.408**	0.326	−4.320	0.000	−2.046	−0.770
Household Size	−2.739	1.796	−1.520	0.127	−6.260	0.782
Age	**0.551**	0.153	3.610	0.000	0.252	0.851
Female	2.655	4.091	0.650	0.516	−5.363	10.673
Non-White	**15.359**	6.451	−2.380	0.017	−28.002	−2.716
4-Year College or Graduate Degree	3.340	4.327	0.770	0.440	−5.142	11.821
Household income $100,000 or higher	**17.535**	4.739	3.700	0.000	8.247	26.824

Note: Marginal effects are calculated with other variables at their mean levels (see Table A-2 for summary statistics). Marginal effects for binary (dummy variables) are for the discrete change from the base level.

**Table 8.** Reported and Measured One-Way Distance and Time Traveled for Last Day Trip

Distance Traveled	Mean	Linearized Std. Error	95% Confidence Interval	
Reported distance (mi)	30.2	1.53	27.18	33.18
Measured distance (mi)	36.8	1.49	33.88	39.73
Reported time (min)	56.4	3.18	50.11	62.61
Measured time (min)	59.0	3.33	52.48	65.57

**Table 9:** Summary of Participation, Time Spent, and Miles Traveled for the Study Region

Description	N	95% Confidence Interval	
Total participants (people 18+)	4,629,453		
Total trips per year	170,498,066	154,316,971	186,679,160
Average hours spent on site per person per day	4.53	4.24	4.82
Average recreation hours per person per year	167	141	194
Total coastal recreation hours per year	772,363,810	723,068,451	821,659,170
Average round trip hours traveled per person per trip	1.79	1.58	1.99
Average round trip hours traveled per person per year	66	53	80
Total travel hours per year	304,578,781	269,169,017	339,988,546
Average round trip miles traveled per person per trip	73.61	67.76	79.46
Average miles traveled per person per year	2,711	2,259	3,204
Total travel miles per year (does not account for multiple people/vehicle)	12,549,623,266	11,552,348,141	13,546,898,391
Average trip expenditure per person per day (2021$)*	$69.68*		
Total estimated trip expenditures per year	$11.88 billion		

* Source: Kosaka and Steinback, 2018. Converted from 2012$ to 2021$ using the Bureau of Labor Statistics Consumer Price Index (CPI).

## Data Availability

The datasets analyzed in this study can be found at https://doi.org/10.5281/zenodo.5807859.

## Footnotes

1 For statistics about individuals, we multiplied household numbers by 1.911, the average number of people over 18 per household in our sampled region.

2 Wildlife viewing includes birding and general wildlife viewing; fishing and hunting includes finfishing, shellfishing, spearfishing, and hunting; board sports includes kiteboarding, windsurfing, paddleboarding, skimboarding, surfing, and boogie boarding; water immersion includes scuba diving, skimboarding, snorkeling, spearfishing, surfing, boogie boarding, swimming, body surfing, tubing, and waterskiing; motorized boating includes jetskiing, motorboating, tubing, and waterskiing; non-motorized boating includes kayaking, canoeing, rowing, and sailing.

3 Information presented is for locations identified as valid for travel cost models (i.e., those that can be located with sufficient precision along the coast).

4 The “303(d) list” is a list of impaired or threatened waters compiled by states, territories, and tribes and submitted to the U.S. Environmental Protection Agency as required by the Clean Water Act (https://www.epa.gov/tmdl).

5 This section reports values for single day trips only, not including those that are part of a multi-day trip.

## APPENDIX

**Table A-1.** Demographics for Web and Paper Survey Respondents, Compared to Study Area Population Demographics

Demographics	Web Survey	Paper Survey	Study Area Population
Male	53%	40%	49%
Female	47%	60%	51%
Median Age	56	64	48
White	80%	94%	82%
High School Education	26%	41%	24%
Bachelor’s Degree	33%	30%	24%
Graduate Degree	33%	27%	17%
Employed	65%	48%	64%
Retired, student, unemployed	35%	52%	34%
Household Income			
$0–99.9k	50%	62%	59%
$100–199.9k	35%	28%	28%
$200k and over	15%	10%	12%

Note: Study area demographics are from US Census 2014–2019 American Community Survey 5-year estimates for the sampled counties (U.S. Census, 2021). Census data weighted by county using the survey base weights.

**Table A-2.** Respondents’ Distance from Coast (miles) by State

State	N	Mean Distance (mi.)	Standard Deviation	Minimum	Maximum
CT	122	4.4	5.1	0.01	23.1
RI	154	1.5	2.5	0.00	14.6
MA	1,059	6.5	9.7	0.00	52.2
NH	102	17.1	10.7	0.13	44.9

**Table A-3.** Percent of Trips From Home States to Destination States

		Destination State				
Home	State	CT	RI	MA	NH	ME
CT	All trips	**56.0%**	20.2%	15.5%	0%	8.3%
	Day trips	**71.4%**	22.2%	1.6%	0%	4.8%
	Overnight trips	**9.5%**	14.3%	57.1%	0%	19.0%

RI	All trips	0%	**86.0%**	12.3%	0.9%	0.9%
	Day trips	0%	**91.1%**	7.9%	1.0%	0%
	Overnight trips	0%	**46.2%**	46.2%	0%	7.7%

MA	All trips	1.3%	4.8%	**80.4%**	4.4%	9.2%
	Day trips	1.6%	3.5%	**87.3%**	3.9%	3.7%
	Overnight trips	0.5%	8.7%	**59.2%**	6.0%	25.5%

NH	All trips	0%	2.7%	18.9%	**58.1%**	20.3%
	Day trips	0%	1.6%	18.0%	**68.9%**	11.5%
	Overnight trips	0%	7.7%	23.1%	**7.7%**	61.5%

**Table A-4.** Participation Rates for Respondents to Web and Paper Surveys

	N	Mean	Linearized Standard Error	95% Confidence Interval	
Web survey	780	77.0%	1.8%	73.6%	80.5%
Paper survey	657	64.5%	2.2%	60.2%	68.8%

**Table A-5.** Mean Days Spent per Year for Respondents to Web and Paper Surveys (Participants Only)*

	N	Mean	Linearized Standard Error	95% Confidence Interval	
Web survey	617	33.6	2.0	29.8	37.5
Paper survey	386	41.8	3.4	35.2	48.4

* Truncated at 95^th^ percentile

**Table A-6.** Summary Statistics for Variables Included in Participation and Effort Models

Variable	N	Mean Value	Standard Deviation	Min	Max
Miles to coast* – all respondents	1,437	6.57	9.55	0.00	52.24
Miles to coast* – non-participants	356	8.53	10.63	0.01	47.70
Miles to coast* – participants	1,081	5.92	9.07	0.00	52.24

Household size – all respondents	1,327	2.36	1.21	1	9
Household size – non-participants	321	2.04	1.14	1	8
Household size – participants	1,006	2.47	1.21	1	9

Age – all respondents	1,295	57.57	16.05	15	103
Age – non-participants	315	62.86	17.41	15	95
Age – participants	980	55.87	15.21	17	103

Female – all respondents	1,330	0.52	0.50	0	1
Female – non-participants	319	0.51	0.50	0	1
Female – participants	1,011	0.52	0.50	0	1

Non-white** – all respondents	1,209	0.07	0.26	0	1
Non-white** – non-participants	298	0.15	0.36	0	1
Non-white** – participants	911	0.05	0.22	0	1

High education*** – all respondents	1,337	0.66	0.47	0	1
High education*** – non-participants	328	0.48	0.50	0	1
High education*** – participants	1,009	0.72	0.45	0	1

High income**** – all respondents	1,008	0.44	0.50	0	1
High income**** – non-participants	249	0.24	0.43	0	1
High income**** – participants	759	0.51	0.50	0	1

Second home***** – all respondents	1,320	0.11	0.31	0	1
Second home***** – non-participants	323	0.04	0.20	0	1
Second home***** – participants	997	0.13	0.34	0	1

Shore activities – all respondents	1,437	0.66	0.47	0	1
Shore activities – participants	1,081	0.88	0.32	0	1

Wildlife viewing – all respondents	1,437	0.26	0.44	0	1
Wildlife viewing – participants	1,081	0.34	0.47	0	1

Fishing or hunting – all respondents	1,437	0.19	0.40	0	1
Fishing or hunting – participants	1,081	0.26	0.44	0	1

Boardsports – all respondents	1,437	0.13	0.33	0	1
Boardsports – participants	1,081	0.17	0.37	0	1

Nonmotorized boating – all respondents	1,437	0.23	0.42	0	1
Nonmotorized boating – participants	1,081	0.31	0.46	0	1

Motorized boating – all respondents	1,437	0.16	0.37	0	1
Motorized boating – participants	1,081	0.22	0.41	0	1

Water immersion – all respondents	1,437	0.47	0.50	0	1
Water immersion – participants	1,081	0.62	0.49	0	1

* Straight line distance from center of residence census block to coast

** Any single or combined race category that does not include white

*** 4-year college degree or graduate degree

**** HH income $100,000 or higher

***** Owns a second home at the coast

**Table A-7.** Logit Participation Model

Variable	Coefficient	Standard Error	T statistic	P value	Confidence Interval	
Distance from residence to coast (mi.)	**−0.029**	0.009	−3.470	0.001	−0.046	−0.013
Household Size	0.102	0.093	1.100	0.271	−0.080	0.284
Age	0.053	0.034	1.541	0.123	−0.014	0.120
Age squared	**−0.001**	0.0003	−2.509	0.012	−0.001	−0.000
Female	−0.022	0.184	−0.118	0.906	−0.382	0.339
Non-white	**−1.923**	0.308	−6.242	0.000	−2.527	−1.319
4-year college degree or graduate degree	**0.875**	0.190	4.602	0.000	0.503	1.248
Household income $100,000 or higher	**0.570**	0.203	2.807	0.005	0.172	0.968
Constant	0.106	0.995	0.110	0.915	−1.844	2.055

Dependent variable: participated or not (1/0)

N = 978 Log pseudolikelihood = −1170567

Wald chi^2^(8) = 125.21 Prob > chi^2^ = 0.0000

Pseudo R^2^ = 0.170

**Table A-8.** Percent of People Who Participated in Activities by Demographic Categories and Distance From Coast

	All	Female	Non-White	Income<$100k	Income >=$100k	Has 2nd Home on Coast	<=2 mi from coast	>2 mi from coast
**N in sample**	1,437	694	72	560	691	147	756	681
Activities on the shore	62.0%	66.7%	35.0%	53.3%	76.3%	78.6%	68.0%	56.8%
Birding, Wildlife Viewing	21.6%	23.4%	6.3%	18.2%	24.7%	31.2%	25.3%	18.5%
Fishing	14.3%	8.1%	6.7%	10.0%	21.0%	26.2%	16.4%	12.5%
Hunting	0.7%	0.4%	0.0%	0.5%	1.2%	0.8%	1.3%	0.2%
Jet skiing	1.6%	1.6%	0.0%	1.0%	2.7%	7.8%	2.5%	0.8%
Kayaking, Canoeing, Rowing	15.4%	16.1%	3.9%	10.1%	26.5%	26.6%	20.3%	11.2%
Kiteboarding, Windsurfing	0.5%	0.3%	0.0%	0.2%	1.1%	0.7%	0.3%	0.6%
Motorboating	11.7%	9.5%	2.1%	7.4%	20.1%	21.9%	15.7%	8.3%
Paddleboarding	5.0%	6.4%	3.9%	3.3%	8.8%	12.7%	7.9%	2.5%
Sailing	7.0%	6.5%	5.5%	4.2%	12.2%	8.1%	10.7%	3.8%
Scuba diving	1.3%	0.9%	0.9%	1.1%	1.3%	2.2%	1.8%	0.8%
Shellfishing	4.5%	3.2%	0.0%	2.4%	7.2%	13.4%	6.6%	2.8%
Skimboarding	1.2%	1.5%	1.6%	0.7%	2.1%	2.0%	1.7%	0.9%
Snorkeling	2.7%	1.9%	0.9%	2.0%	3.9%	2.2%	3.2%	2.3%
Spearfishing	0.1%	0.0%	0.0%	0.2%	0.0%	0.0%	0.3%	0.0%
Surfing, Boogie boarding	7.6%	8.6%	3.0%	5.1%	12.4%	11.9%	9.0%	6.5%
Swimming, Body surfing	42.5%	44.6%	20.7%	35.1%	58.4%	58.7%	44.9%	40.4%
Tubing, Waterskiing	2.4%	2.1%	0.0%	1.3%	5.0%	7.7%	3.1%	1.9%
Wading	34.8%	39.4%	13.3%	27.0%	49.5%	44.4%	35.9%	33.8%
Other	1.0%	1.3%	0.0%	0.8%	1.1%	1.2%	1.5%	0.5%
Did not participate	28.8%	28.1%	59.1%	37.4%	15.2%	10.7%	23.6%	33.4%

**Table A-9.** Truncated Negative Binomial Regression of Total Days per Year

Variable	Coefficient	Standard Error	T Statistic	P Value	Confidence Interval	Variable
Distance from residence to coast (mi.)	**−0.045**	0.010	−4.443	0.0000	−0.065	−0.025
Household Size	−0.087	0.056	−1.572	0.1160	−0.196	0.022
Age	**0.059**	0.027	2.166	0.0303	0.006	0.112
Age squared	−0.0004	0.0003	−1.525	0.1273	−0.001	0.0001
Female	0.085	0.131	0.650	0.5160	−0.172	0.342
Nonwhite	−0.648	0.363	−1.785	0.0742	−1.359	0.063
4-year college or graduate degree	0.109	0.145	0.752	0.4521	−0.175	0.392
Household income $100,000 or higher	**0.541**	0.140	3.863	0.0001	0.266	0.815
Constant	**1.645**	0.709	2.321	0.0203	0.256	3.035

ln(alpha)	0.786	0.098			0.594	0.977
Alpha	2.194	0.214			1.812	2.656

Dependent variable: days spent in last 12 months (truncated at 365)

N = 700 Log Pseudolikelihood = −7073729.6

Wald chi^2^(8) = 66.91 Prob > chi^2^ = 0.0000

Pseudo R^2^ = 0.016

**Table A-10.** Truncated Negative Binomial Model of Days per Year, Model 2^6^

Variable	Coefficient	Standard Error	T Statistic	P Value	Confidence Interval	
Distance from residence to coast (mi.)	**−0.048**	0.007	−6.474	0.000	−0.062	−0.033
Household Size	−0.099	0.054	−1.841	0.066	−0.204	0.006
Age	0.055	0.029	1.872	0.061	−0.003	0.112
Age squared	−0.0003	0.0003	−1.077	0.282	−0.001	0.0003
Female	**0.359**	0.134	2.681	0.007	0.097	0.622
Nonwhite	−0.253	0.419	−0.603	0.547	−1.074	0.569
4-year college or graduate degree	−0.027	0.148	−0.180	0.857	−0.317	0.263
Household income $100,000 or higher	**0.544**	0.148	3.678	0.0002	0.254	0.834
Second home at coast	0.334	0.192	1.735	0.083	−0.043	0.711
Activities on the shore	−0.078	0.271	−0.288	0.774	−0.608	0.453
Birding, wildlife viewing	**0.546**	0.126	4.333	0.000	0.299	0.793
Fishing, shellfishing, hunting	**0.479**	0.151	3.163	0.002	0.182	0.775
Board sports	**0.476**	0.178	2.681	0.007	0.128	0.824
Non-motorized boating	0.233	0.144	1.620	0.105	−0.049	0.515
Motorized boating	0.212	0.158	1.340	0.180	−0.098	0.521
Water immersion	0.273	0.167	1.636	0.102	−0.054	0.599
Constant	0.903	0.784	1.15	0.249	−0.633	2.439

/lnalpha	0.542	0.096			0.353	0.730
alpha	1.719	0.165			1.424	2.076

Dependent variable: days spent in last 12 months (truncated at 365)

N = 695 Log Pseudolikelihood = −6841750.3

Wald chi^2^(16) = 236.27 Prob > chi^2^ = 0.0000

Pseudo R^2^ = 0.034

**Table A-11.** Marginal Effects of Respondent Characteristics on Days Spent Recreating, Model 2^7^

Variable	Marginal Effect	Standard Error	z	P>|z|	95% Confidence Interval	
Distance from residence to coast (mi.)	**−1.327**	0.242	−5.480	0.000	−1.802	−0.853
Household Size	−2.737	1.554	−1.760	0.078	−5.784	0.309
Age	**0.621**	0.142	4.390	0.000	0.344	0.899
Female	**9.870**	3.821	2.580	0.010	2.382	17.358
Non-White	−6.256	9.275	−0.670	0.500	−24.436	11.923
4-year college or graduate degree	−0.743	4.147	−0.180	0.858	−8.871	7.385
Household income $100,000 or higher	**15.615**	4.745	3.290	0.001	6.316	24.914
Second home at coast	10.706	7.037	1.520	0.128	−3.086	24.498
Activities on the shore	−2.159	7.529	−0.290	0.774	−16.915	12.598
Birding, wildlife viewing	**15.143**	3.441	4.400	0.000	8.398	21.887
Fishing, shellfishing, hunting	**13.272**	4.240	3.130	0.002	4.961	21.582
Board sports	**13.199**	5.009	2.630	0.008	3.381	23.017
Non-motorized boating	6.467	3.995	1.620	0.105	−1.363	14.297
Motorized boating	5.869	4.339	1.350	0.176	−2.635	14.372
Water immersion	7.556	4.540	1.660	0.096	−1.342	16.454

Note: dy/dx for factor levels is the discrete change from the base level.

**Table A-12.** OLS Regression of Time Spent in Hours for Day Trips

Variable	Coefficient	Standard Error	T Statistic	P Value	Confidence Interval	
Distance from residence to coast (mi.)	**0.039**	0.012	3.360	0.001	0.016	0.062
High effort*	**−0.720**	0.252	−2.860	0.004	−1.215	−0.225
Shore activities	−0.693	0.381	−1.820	0.069	−1.440	0.054
Wildlife viewing	**−0.517**	0.242	−2.130	0.033	−0.993	−0.042
Fishing, hunting	0.632	0.285	2.210	0.027	0.072	1.193
Boardsports	0.227	0.337	0.670	0.501	−0.4341	0.888
Nonmotorized boating	−0.278	0.262	−1.060	0.290	−0.793	0.237
Motorboating	**0.736**	0.275	2.680	0.008	0.196	1.276
Water Immersion	**0.521**	0.195	2.670	0.008	0.138	0.904
Sense of place score**	**0.019**	0.007	2.670	0.008	0.005	0.033
Constant	**3.347**	0.404	8.280	0.000	2.553	4.140

Dependent variable: hours spent on last day trip

N=761

F (10, 750) = 7.74 R-squared = 0.1149

Prob >F = 0.0000 Root MSE = 2.2382

* More than 78 days per year

** The sense of place score is a measure that represents the emotional attachment to a specific place.

Figures A-1 through A-8 below show the locations visited on the last trip by people who engaged in different categories of activities.
