# Supplementary material for: Coastal Recreation in Southern New England: Results from a Regional Survey
Source: J Ocean Coast Econ. Author manuscript; Available in PMC 2023 Aug 1. (PMC9580342; doi:10.15351/2373-8456.1152)
Supplement: Supplement1 [file NIHMS1832734-supplement-Supplement1.pdf]

United States
Environmental Protection
Agency

OMB Control Number 2080-0084
Expires 04/30/2021

New England Coastal Water Quality and Recreation Survey

This survey asks for your opinions on coastal water quality in New England and how you use coastal areas for recreation. Your answers to this survey will help inform decisions to improve and protect coastal water quality.

We want to hear from everyone. Even if you do not participate in coastal water recreation or visit coastal New England, some questions will apply to you.

The survey should take you around 15 minutes to complete. There are no wrong answers, but please read each question carefully. Please return your completed survey in the provided postage-paid envelope. Thank you for your help!

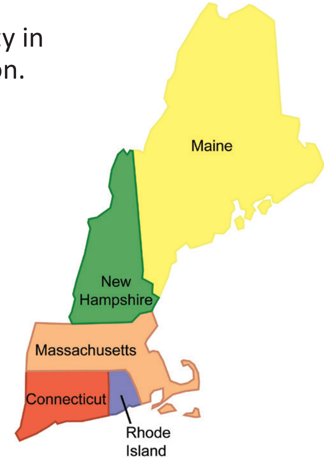

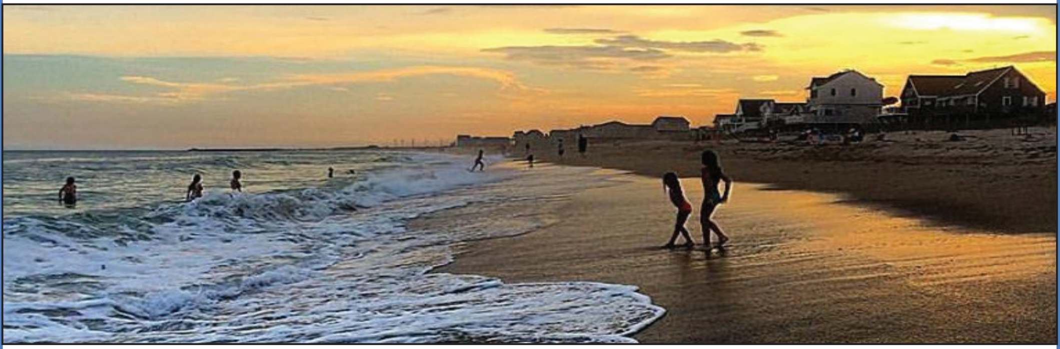

Your response is important!

All responses will be kept confidential. Response to this survey is voluntary. Send comments on any aspect of this survey to Recreation Survey, Atlantic Ecology Division, U.S. Environmental Protection Agency, 27 Tarzwell Drive, Narragansett, Rhode Island 02882.

EPA Form Number 6000-03 (April 2018)

1019764

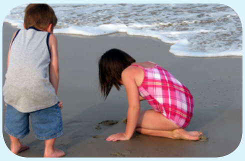

Section 1: Your Saltwater Recreation in New England

(Maine, New Hampshire, Massachusetts, Rhode Island, Connecticut)

1.1. Which **saltwater** activities have you participated in during the last 12 months in **New England**? Please think only about activities on, in, or near the ocean, bays, estuaries, salt ponds, and other salty or brackish tidal waters. (Select all that apply)

- O Activities on the shore, not in the water (walking, dog walking, sitting, sunbathing, games, beach-combing, picnicking, sandcastles, kite flying, photography, etc.)
- O Birding/wildlife viewing
- O Fishing
- O Hunting
- O Jetskiing
- O Kayaking/canoeing/rowing
- O Kiteboarding/windsurfing
- O Motorboating
- O Paddleboarding
- O Sailing
- O Scuba diving
- O Shellfishing
- O Skimboarding
- O Snorkeling
- O Spearfishing
- O Surfing/boogie boarding
- O Swimming/body surfing
- O Tubing/waterskiing
- O Wading
- O Other saltwater activities, please list: ______________________________
- O I did not participate in saltwater recreation in New England in the past 12 months.

If you checked any of the saltwater activities above or wrote in a saltwater activity, please continue on to Question 1.2.

If you did NOT participate in saltwater recreation in the past 12 months, please SKIP to Section 4, page 16.

1.2. In the last 12 months, how often did you participate in **saltwater recreation in New England**? This means any activities on, in, or near coastal New England waters. Consider any time when you spent a half hour or more.
(Fill in one number for each season – **either** times per week or per season.)

Summer (June, July, Aug)	☐☐☐	times per **week**	OR	☐☐☐	per **season**
Fall (Sept, Oct, Nov)	☐☐☐	times per **week**	OR	☐☐☐	per **season**
Winter (Dec, Jan, Feb)	☐☐☐	times per **week**	OR	☐☐☐	per **season**
Spring (March, April, May)	☐☐☐	times per **week**	OR	☐☐☐	per **season**

1019764

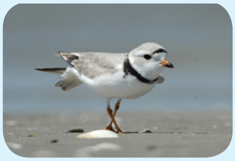

Section 2: Your Most Recent Saltwater Recreation in New England

This section asks about the **last time** you participated in saltwater recreation in New England, **on a single day OR as part of an overnight trip.**

A **single day** means a day when you left from and returned to your primary or seasonal home on the same day to participate in saltwater recreation activities for a half hour or more.

An **overnight trip** or vacation is any trip in New England that included saltwater recreation when you stayed overnight somewhere that was not your primary or seasonal home.

2.1. Was the last time you participated in saltwater recreation in New England part of a single day or overnight trip?

- O Single day → **Continue to Section 2A below.**
- O Overnight trip → **Skip to Section 2B on Page 7.**

Section 2A: Your Most Recent SINGLE DAY of Saltwater Recreation in New England

(A day when you left from and returned to your home on the same day.)

2.2.A. When was the **last time** you participated in **saltwater** recreation in New England on a single day?

a. Month:

O	O	O	O	O	O	O	O	O	O	O	O
J	F	M	A	M	J	J	A	S	O	N	D

b. Year: ☐☐☐☐ (YYYY)

c. Was it on a: O Weekend/holiday **OR** O Weekday?

2.3.A. Where did you go?

O Maine O New Hampshire O Massachusetts O Rhode Island O Connecticut

City or town: ____
(If you don't know the city or town, give the approximate part of the state.)

Name of place: ______________________________________
(If possible, please be more specific than town. Examples: beach name, street name, marina or park name.)

1019764

2.4.A. Why did you choose that place? (Please describe)

2.5.A. How many times have you gone to that place in the last 12 months? (Select one)

- O 1 time
- O 2-5 times
- O 6-15 times
- O 16-30 times
- O 31-50 times
- O More than 50 times

2.6.A. What type of transportation did you use to travel there? (Select all that apply)

- O Car/truck
- O Bicycle
- O Ferry
- O Private boat
- O Public transportation
- O Walked
- O Other? ______________________________

2.7.A. How far did you travel (**one way**) from your home to get there? Your best estimate is fine.

One way distance: ☐☐☐ miles

Time it took to get there, one way (including traffic): ☐☐ hours ☐☐ minutes

2.8.A. How many people went with you (**including yourself**)?

☐☐ adults ☐☐ children (under 18)

2.9.A. How long did you spend there on that day?

☐☐ hours ☐☐ minutes

2.10.A. Did you, or someone you went with, use a parking pass for that place?

- O No
- O Yes → Cost of pass: $ ☐☐☐.☐☐ Pass used how many times per year: ☐☐☐
- O Don't know

2.11.A. Did you pay a daily parking fee?

- O No
- O Yes → How much? $ ☐☐☐.☐☐

1019764

2.12.A. Which **saltwater** activities did you participate in on that day? **(Select all that apply)**

- O Activities on the shore, not in the water (walking, dog walking, sitting, sunbathing, games, beach-combing, picnicking, sandcastles, kite flying, photography, etc.)
- O Birding/wildlife viewing
- O Fishing
- O Hunting
- O Jetskiing
- O Kayaking/canoeing/rowing
- O Kiteboarding/windsurfing
- O Motorboating
- O Paddleboarding
- O Sailing
- O Scuba diving
- O Shellfishing
- O Skimboarding
- O Snorkeling
- O Spearfishing
- O Surfing/boogie boarding
- O Swimming/body surfing
- O Tubing/waterskiing
- O Wading
- O Other saltwater activities, please list: ___________________________

2.13.A. Which was **your most important activity** on that day? **(Select one)**

- O Activities on the shore, not in the water (walking, dog walking, sitting, sunbathing, games, beach-combing, picnicking, sandcastles, kite flying, photography, etc.)
- O Birding/wildlife viewing
- O Fishing
- O Hunting
- O Jetskiing
- O Kayaking/canoeing/rowing
- O Kiteboarding/windsurfing
- O Motorboating
- O Paddleboarding
- O Sailing
- O Scuba diving
- O Shellfishing
- O Skimboarding
- O Snorkeling
- O Spearfishing
- O Surfing/boogie boarding
- O Swimming/body surfing
- O Tubing/waterskiing
- O Wading
- O Other saltwater activities, please list: ___________________________

2.14.A. How much contact with the water did you have that day? (Select one)

O Did not get wet at all   O Got sprayed   O Waded   O Got most or all of my body wet

Water quality describes how clean or dirty the water is - specifically its quality for recreation such as swimming, fishing or shellfishing, boating, and other activities. Water quality can affect marine life, your health, and your enjoyment of recreation.

2.15.A. How do you think the water quality usually is, on average, at that place (Question 2.3.A, page 2)? (Select a number)

**Worst possible quality:** may have bad odor, oil, raw sewage; unhealthy for plant and animal life.	1	2	3	4	5	6	7	8	9	10	**Best possible quality:** clear, safe for all activities, never has closures, healthy for plant and animal life.
	O	O	O	O	O	O	O	O	O	O	

2.16.A. How sure were you about your water quality rating in question 2.15.A?

O Not very sure   O Somewhat sure   O Sure   O Very sure

1019764

2.17.A. How would you rate the usual condition of the water at that place, for the following aspects of water quality? (Fill in one circle for each aspect)

Aspects	Worst Quality	1	2	3	4	5	6	7	8	9	10	Best Quality	Don't Know
Seaweed	Wide spread	○	○	○	○	○	○	○	○	○	○	None	○
Algae/scum	Wide spread	○	○	○	○	○	○	○	○	○	○	Absent	○
Bacteria	Excessive	○	○	○	○	○	○	○	○	○	○	Absent	○
Clarity of the water	Murky	○	○	○	○	○	○	○	○	○	○	Clear	○
Mucky bottom condition	Very mucky	○	○	○	○	○	○	○	○	○	○	Not at all mucky	○
Oil or gas sheen	Common	○	○	○	○	○	○	○	○	○	○	Rare	○
Smell/odor	Bad odor	○	○	○	○	○	○	○	○	○	○	Fresh	○
Trash in water	Common	○	○	○	○	○	○	○	○	○	○	Rare	○

2.18.A. Overall, how would you rate the usual **quality of the water** (how clean or dirty the water is) at that place for each of these activities? (Fill in one circle for each row, even if you don't participate in all of these activities)

Activity	Poor	Okay	Good	Don't Know or Not Applicable
Canoeing/kayaking/paddleboarding	○	○	○	○
Eating fish caught in the water	○	○	○	○
Shellfishing	○	○	○	○
Fishing	○	○	○	○
Motorboating/sailing	○	○	○	○
Picnicking and other activities on the shore	○	○	○	○
Scenic beauty/enjoyment	○	○	○	○
Snorkeling/scuba diving	○	○	○	○
Swimming	○	○	○	○
Wading	○	○	○	○

1019764

2.19.A. Thinking about the place you visited most recently for saltwater recreation (Question 2.3.A, page 2), please indicate how much you agree or disagree with the following statements. (Fill in one circle for each statement)

	Strongly Disagree			⟷			Strongly Agree
Many important memories are tied to that place.	○	○	○	○	○	○	○
The recreational activities that I pursue at that place say a lot about who I am.	○	○	○	○	○	○	○
I have no particular love for that place compared to other areas.	○	○	○	○	○	○	○
I get more satisfaction out of visiting that place than any other recreation place.	○	○	○	○	○	○	○
That place means a lot to me.	○	○	○	○	○	○	○
I identify with the physical landscape of that place.	○	○	○	○	○	○	○
I feel happiest when I'm at that place.	○	○	○	○	○	○	○
I am very attached to that place.	○	○	○	○	○	○	○
That place provides value to me that I can't obtain elsewhere.	○	○	○	○	○	○	○

2.20.A. What is important to you about that place, if anything?

This is the end of Section 2 Part A about the last time you participated in saltwater recreation on a single day. Please **SKIP to Section 3 on Page 14.**

1019764

Section 2B: Your Most Recent OVERNIGHT TRIP that Included Saltwater Recreation in New England

(An **overnight trip** or vacation is when you stayed overnight somewhere that was not your permanent or seasonal home.)

2.2.B. When was the **last time** you took an overnight trip in New England that included **saltwater** recreation?

a. What month: ○ J ○ F ○ M ○ A ○ M ○ J ○ J ○ A ○ S ○ O ○ N ○ D

b. Year: ☐☐☐☐ (YYYY)

2.3.B. Where did you stay overnight?

○ Maine ○ New Hampshire ○ Massachusetts ○ Rhode Island ○ Connecticut

City or town: ______________________________

(If you don't know the city or town, give the approximate part of the state.)

2.4.B. In the past 5 years, how many times have you gone on vacation or an overnight trip to that place? ☐☐☐ times

2.5.B. What type of transportation did you use to travel there? (Select all that apply)

○ Airplane ○ Ferry ○ Recreational vehicle or camper

○ Bicycle ○ Private boat ○ Tour bus

○ Car/truck ○ Public transportation ○ Other? ____________________

2.6.B. How far did you travel (**one way**) from your home to get to the place where you stayed overnight (not including stops along the way such as shopping or sightseeing)? Your best estimate is fine.

One way distance: ☐☐☐ miles

Time it took to get there, one way (including traffic): ☐☐ hours ☐☐ minutes

2.7.B. How many people went with you (**including yourself**)?

☐☐ adults ☐☐ children (under 18)

1019764

2.8.B. How many nights did you stay away from home on that trip? [ ][ ][ ] nights

2.9.B. Where did you stay on that vacation or overnight trip? (Select all that apply)

- ○ Rental home or condo
- ○ Your second home or condo
- ○ Friend or relative's house or condo
- ○ Hotel/resort/bed & breakfast
- ○ Campsite
- ○ Other? ___________________________

2.10.B. What was the total cost for lodging for the trip?

$ [ ][ ][ ][ ][ ] . [ ][ ]

2.11.B. How important was saltwater recreation as a reason for taking that trip?

- ○ The most important activity
- ○ One of the most important activities
- ○ One of several activities
- ○ A minor activity
- ○ Not an important activity

2.12.B. Which of these saltwater activities did you participate in on your overnight trip? **(Select all that apply)**

- ○ Activities on the shore, not in the water (walking, dog walking, sitting, sunbathing, games, beach-combing, picnicking, sandcastles, kite flying, photography, etc.)
- ○ Birding/wildlife viewing
- ○ Fishing
- ○ Hunting
- ○ Jetskiing
- ○ Kayaking/canoeing/rowing
- ○ Kiteboarding/windsurfing
- ○ Motorboating
- ○ Paddleboarding
- ○ Sailing
- ○ Scuba diving
- ○ Shellfishing
- ○ Skimboarding
- ○ Snorkeling
- ○ Spearfishing
- ○ Surfing/boogie boarding
- ○ Swimming/body surfing
- ○ Tubing/waterskiing
- ○ Wading
- ○ Other saltwater activities, please list: _______________________________________

1019764

2.13.B. Which was **your most important activity** on that trip? **(Select one)**

- O Activities on the shore, not in the water (walking, dog walking, sitting, sunbathing, games, beach-combing, picnicking, sandcastles, kite flying, photography, etc.)
- O Birding/wildlife viewing
- O Fishing
- O Hunting
- O Jetskiing
- O Kayaking/canoeing/rowing
- O Kiteboarding/windsurfing
- O Motorboating
- O Paddleboarding
- O Sailing
- O Scuba diving
- O Shellfishing
- O Skimboarding
- O Snorkeling
- O Spearfishing
- O Surfing/boogie boarding
- O Swimming/body surfing
- O Tubing/waterskiing
- O Wading
- O Other saltwater activities, please list: ______________________________

2.14.B. How many days of that trip did you participate in **your most important activity**?

☐☐☐ days

These next questions ask about a **single time** that you participated in **your most important activity** during your overnight trip or vacation. Some of these questions may be similar to questions you've already answered, but please answer all the questions.

2.15.B. Please recall only the **LAST TIME** that you did your **most important** activity from Question 2.13.B, on that vacation or overnight trip. Where did you go for that activity? (This may or may not be the same location where you stayed)

O Maine O New Hampshire O Massachusetts O Rhode Island O Connecticut

City or town: ______________________________
(If you don't know the city or town, give the approximate part of the state.)

Name of place: ______________________________
(If possible, be more specific than town. Examples: beach name, street name, marina or park name.)

2.16.B. Why did you choose that place? (Please describe)

2.17.B. In the past 12 months, how many times have you gone to the place you listed previously (2.15.B)? (Select one)

- ○ 1 time
- ○ 2-5 times
- ○ 6-15 times
- ○ 16-30 times
- ○ 31-50 times
- ○ More than 50 times

2.18.B. What type of transportation did you use to travel there from where you stayed overnight? (Select all that apply)

- ○ Car/truck
- ○ Bicycle
- ○ Ferry
- ○ Private boat
- ○ Public transportation
- ○ Walked
- ○ Other? ______________________________

2.19.B. How far did you travel (**one way**) from where you stayed overnight to get to the place you listed above (2.15.B)? Your best estimate is fine.

One way distance: ☐☐☐ miles

Time it took to get there that day (including traffic): ☐☐ hours ☐☐ minutes

2.20.B. How many people went with you to the place you listed above (2.15.B) (**including yourself**)?

☐☐ adults ☐☐ children (under 18)

2.21.B. How long did you spend at that place on that day?

☐☐☐ hours ☐☐ minutes

2.22.B. Did you, or someone you went with, use a parking pass for that place?

○ No ○ Yes → Cost of pass: $☐☐☐.☐☐ Pass used how many times per year: ☐☐☐

○ Don’t know

2.23.B. Did you pay a daily parking fee?

○ No ○ Yes → How much? $☐☐☐.☐☐

2.24.B. How much contact with the water did you have that day? (Select one)

O Did not get wet at all   O Got sprayed   O Waded   O Got most or all of my body wet

Water quality describes how clean or dirty the water is - specifically its quality for recreation such as swimming, fishing or shellfishing, boating, and other activities. Water quality can affect marine life, your health, and your enjoyment of recreation.

2.25.B. How do you think the water quality usually is, on average, at that place (Question 2.15.B, page 9)? (Select a number)

**Worst possible quality:** may have bad odor, oil, raw sewage; unhealthy for plant and animal life.	1	2	3	4	5	6	7	8	9	10	**Best possible quality:** clear, safe for all activities, never has closures, healthy for plant and animal life.
	O	O	O	O	O	O	O	O	O	O	

2.26.B. How sure were you about your water quality rating in question 2.25.B?

O Not very sure   O Somewhat sure   O Sure   O Very sure

2.27.B. How would you rate the usual condition of the water, at that place, for the following aspects of water quality? (Fill in one circle for each aspect)

Aspects	Worst Quality	1	2	3	4	5	6	7	8	9	10	Best Quality	Don't Know
Seaweed	Wide spread	O	O	O	O	O	O	O	O	O	O	None	O
Algae/scum	Wide spread	O	O	O	O	O	O	O	O	O	O	Absent	O
Bacteria	Excessive	O	O	O	O	O	O	O	O	O	O	Absent	O
Clarity of the water	Murky	O	O	O	O	O	O	O	O	O	O	Clear	O
Mucky bottom condition	Very mucky	O	O	O	O	O	O	O	O	O	O	Not at all mucky	O
Oil or gas sheen	Common	O	O	O	O	O	O	O	O	O	O	Rare	O
Smell/odor	Bad odor	O	O	O	O	O	O	O	O	O	O	Fresh	O
Trash in water	Common	O	O	O	O	O	O	O	O	O	O	Rare	O

1019764

2.28.B. Overall, how would you rate the usual **quality of the water,** (how clean or dirty the water is) at that place for each of these activities? (Fill in one circle for each row, even if you don't participate in all of these activities)

**Activity**	**Poor**	**Okay**	**Good**	**Don't Know or Not Applicable**
Canoeing/kayaking/paddleboarding	○	○	○	○
Eating fish caught in the water	○	○	○	○
Shellfishing	○	○	○	○
Fishing	○	○	○	○
Motorboating/sailing	○	○	○	○
Picnicking and other activities on the shore	○	○	○	○
Scenic beauty/enjoyment	○	○	○	○
Snorkeling/scuba diving	○	○	○	○
Swimming	○	○	○	○
Wading	○	○	○	○

2.29.B. Thinking about the place you visited most recently for saltwater recreation (from Question 2.15.B, page 9), please indicate how much you agree or disagree with the following statements. (Fill in one circle for each statement)

	Strongly Disagree			⟷			Strongly Agree
Many important memories are tied to that place.	○	○	○	○	○	○	○
The recreational activities that I pursue at that place say a lot about who I am.	○	○	○	○	○	○	○
I have no particular love for that place compared to other areas.	○	○	○	○	○	○	○
I get more satisfaction out of visiting that place than any other recreation place.	○	○	○	○	○	○	○
That place means a lot to me.	○	○	○	○	○	○	○
I identify with the physical landscape of that place.	○	○	○	○	○	○	○
I feel happiest when I'm at that place.	○	○	○	○	○	○	○
I am very attached to that place.	○	○	○	○	○	○	○
That place provides value to me that I can't obtain elsewhere.	○	○	○	○	○	○	○

2.30.B. What is important to you about that place, if anything?

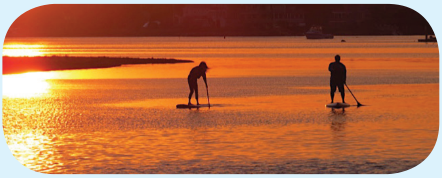

Section 3: Other Places for Saltwater Recreation

This section asks about places you've been in New England with the **WORST** and **BEST** water quality.

3.1. Of the places you've visited for saltwater recreation in **New England** in the last 5 years, which place had the **WORST** water quality?

○ Maine ○ New Hampshire ○ Massachusetts ○ Rhode Island ○ Connecticut

City or Town: ______________________________

(If you don't know the city or town, give the approximate part of the state.)

Name of place: ______________________________

(If possible, be more specific than town. Examples: beach name, street name, marina or park name.)

3.2. How many times have you gone to that place in the last **12 months**? (Select one)

○ 0 times ○ 1 time ○ 2-5 times ○ 6-15 times

○ 16-30 times ○ 31-50 times ○ More than 50 times

3.3. How do you think the water quality usually is, on average, at that place? (Select a number)

Worst possible quality: may have bad odor, oil, raw sewage; unhealthy for plant and animal life.

1	2	3	4	5	6	7	8	9	10
○	○	○	○	○	○	○	○	○	○

Best possible quality: clear, safe for all activities, never has closures, healthy for plant and animal life.

3.4. What about that place made you think it had the **WORST** water quality?

1019764

3.5. Of the places you've visited for saltwater recreation in **New England** in the last 5 years, which place had the **BEST** water quality?

○ Maine ○ New Hampshire ○ Massachusetts ○ Rhode Island ○ Connecticut

City or Town: ______________________________

(If you don't know the city or town, give the approximate part of the state.)

Name of place: ______________________________

(If possible, be more specific than town. Examples: beach name, street name, marina or park name.)

3.6. How many times have you gone to that place in the last **12 months**? (Select one)

○ 0 times ○ 1 time ○ 2-5 times ○ 6-15 times

○ 16-30 times ○ 31-50 times ○ More than 50 times

3.7. How do you think the water quality usually is, on average, at that place? (Select a number)

**Worst possible quality:** may have bad odor, oil, raw sewage; unhealthy for plant and animal life.	1	2	3	4	5	6	7	8	9	10	**Best possible quality:** clear, safe for all activities, never has closures, healthy for plant and animal life.
	○	○	○	○	○	○	○	○	○	○	

3.8. What about that place made you think it had the **BEST** water quality?

3.9. What is the farthest you would travel on a single day (**one way**), leaving from and returning to your home, for saltwater recreation?

One way distance: ☐☐☐ miles

One way time: ☐☐ hours ☐☐ minutes

3.10. If a beach is closed for swimming because of bacterial pollution on a day you are thinking of going, would you…? (Select one)

○ Still go ○ Not go to the beach at all ○ Go to a different beach

1019764

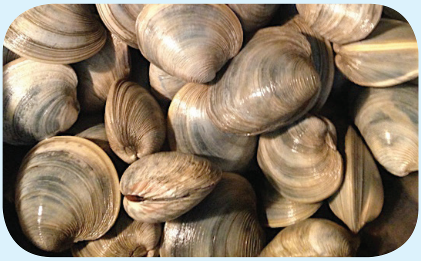

Section 4: Your Opinions on Coastal Water Quality in New England

Coastal water quality is specific to the ocean, bays, estuaries, salt ponds, and other salty or brackish tidal waters in New England. **Water quality** is used to describe how clean or dirty the water is.

4.1. How concerned are you about the following coastal water quality issues in New England? (Fill in one circle in each row)

	Not at all Concerned	Slightly Concerned	Moderately Concerned	Very Concerned	Don't Know
Beaches closed for swimming	○	○	○	○	○
Contaminated fish	○	○	○	○	○
Shellfish closures	○	○	○	○	○
Too much seaweed/algae	○	○	○	○	○
Fish dying from lack of oxygen	○	○	○	○	○
Loss of desirable fish/wildlife	○	○	○	○	○
Unpleasant odor in coastal areas	○	○	○	○	○
Reduced beauty of coastal areas	○	○	○	○	○
Reduced quality of water activities	○	○	○	○	○
Reduced water clarity	○	○	○	○	○

1019764

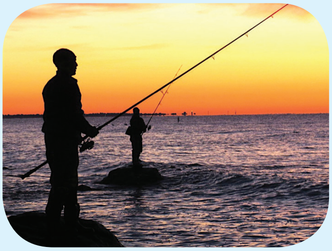

Section 5: About Your Household

The following questions are a way to make sure that we understand the values and opinions of all types of people within New England.

All answers are strictly confidential and the information will only be used to report comparisons among people. We will never identify individuals or households with their responses.

5.1. What is the zip code of your primary home? Zip code: ☐☐☐☐☐

5.2. If you work outside the home, what is the zip code or name of the town and state where you work?

Zip code: ☐☐☐☐☐ **OR** Name of town/city, state: ______________________

5.3. When you chose your primary home, how important was being close to saltwater for recreation? (Select one)

- O Not at all important
- O A minor reason
- O Somewhat important
- O Extremely important
- O The main reason

5.4. How many people (including yourself) live in your household?

☐☐ adults ☐☐ children (under 18)

5.5. What is your gender? O Male O Female O Other

5.6. What year were you born? ☐☐☐☐ (YYYY)

5.7. Do you identify as Hispanic or Latino? O Yes O No O I prefer not to answer

5.8. What race or races do you consider yourself to be? (Select one or more)

- O American Indian or Alaska Native
- O White
- O Asian
- O Black or African American
- O Native Hawaiian or Other Pacific Islander

5.9. What is the highest level of education you have completed? (Select one)

- O Some high school
- O Some college
- O 4-year college degree
- O High school diploma/GED
- O Trade/technical training
- O Graduate degree

5.10. What is your current employment status? (Select one)

- O Employed full-time
- O Unemployed
- O Retired
- O Employed part-time
- O Full-time student
- O Disabled
- O Self-employed
- O Full-time homemaker

5.11. If you are currently employed, how many hours per week do you typically work?

☐☐ hours per week

5.12. If you are currently employed, do you have the option of working additional hours to increase your total income?

O No O Yes → What would your hourly wage be for the extra hours? $☐☐☐.☐☐ / hour

5.13. What was your household income (before taxes) in 2017?

- O $19,999 or less
- O $60,000 - $99,999
- O $200,000 or more
- O $20,000 - $39,999
- O $100,000 - $149,999
- O $40,000 - $59,999
- O $150,000 - $199,999

5.14. Do you own a second home on or near the New England coast?

O No O Yes → What is the zip code or name of town & state where your second home is located?

Zip code: ☐☐☐☐☐ **OR** Name of town/city, state: ________________________________

5.15. How much time do you spend in your second home each year?

- O Seasonal (spend most or all of a season)
- O A week or more but less than a whole season
- O Weekends (spend many or most weekends)
- O Occasional use

1019764

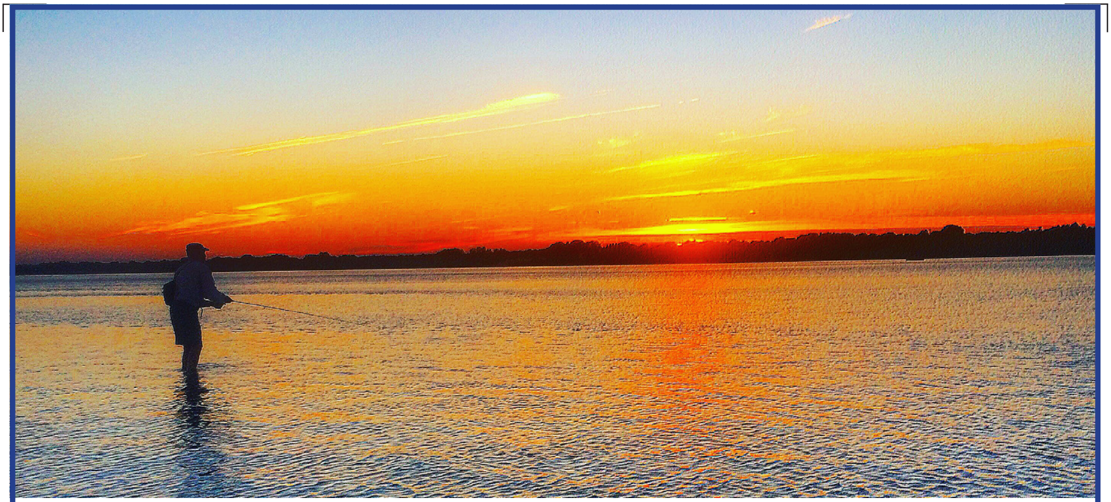

Thank you for taking the time to tell us about your saltwater recreation and opinions about water quality in coastal New England! If you have any additional thoughts and comments about any of the topics or the survey itself, please share them here.

If you would like more information about how you can help improve the water in your coastal region, please visit this website below.
http://www.epa.gov/learn-issues/learn-about-water

The public reporting and recordkeeping burden for this collection of information is estimated to average 15 minutes per response. Send comments on the Agency's need for this information, the accuracy of the provided burden estimates, and any suggested methods for minimizing respondent burden, including through the use of automated collection techniques, to the Director, Collection Strategies Division, U.S. Environmental Protection Agency (2822T), 1200 Pennsylvania Ave., NW, Washington, D.C. 20460. Include the OMB control number in any correspondence. Do not send the completed survey to this address.

Recycled/Recyclable
Printed with vegetable-based ink on paper that contains a minimum of 50% post-consumer fiber content processed chlorine free

1019764
