# Supplementary material for: Coastal Recreation in Southern New England: Results from a Regional Survey
Source: J Ocean Coast Econ. Author manuscript; Available in PMC 2023 Aug 1. (PMC9580342; doi:10.15351/2373-8456.1152)
Supplement: Supplement2 [file NIHMS1832734-supplement-Supplement2.pdf]

Supplementary Information for Coastal Recreation in Southern New England: Results from a Regional Survey

Marisa J. Mazzotta[1§], Nathaniel H. Merrill[1], Kate K. Mulvaney[1]

Procedures for Incorporating Coastal and Shoreline Attributes

THE SHORELINE

The land directly abutting coastal waters in New England varies widely, from fully urbanized in major cities such as Boston and Providence, to the remote, sandy National Seashore. To capture the type of shoreline and to develop a set of shoreline segments to define possible coastal access points, we used the National Oceanic and Atmospheric Administration's (NOAA) Environmental Sensitivity Index (ESI) maps and data (NOAA 2021). Originally designed to gauge the risk from oil spills to the natural and human-use resources of the coastal area, NOAA's national dataset segments the shoreline by type (e.g., sandy, rocky, vegetated, or armored) and by whether it is exposed to or sheltered from wave energy.

Figure 1 illustrates the process of delineating shoreline segments and water segments and linking them. We merged the ESI GIS data for each of the New England states into a single dataset and first simplified the geography by removing any lines that were not categorized as shoreline, which includes docks, piers, jetties, groins, and breakwaters. For our purposes, keeping these features led to undesirable segmentation of the shoreline. We later added these features back as attributes. For each shoreline segment, we recoded the ESI "general shoreline types" to the more general "NEWQ" (New England Water Quality) categories of armored, rocky and steep, beach, or vegetated (see Table 1 for full crosswalk). The NOAA data included up to 3 shoreline types for each segment, representing landward to seaward types. Their simplified "general_symbol" types selected the most environmentally sensitive of these. For our purposes, we were most interested in

[1] *U.S. Environmental Protection Agency, Office of Research and Development, Atlantic Coastal Environmental Sciences Division, 27 Tarzwell Drive, Narragansett, Rhode Island, USA, 02882.*

[§] *Corresponding author, mazzotta.marisa@epa.gov, 401-782-3026*

the type of shoreline access for recreation, so created our crosswalk to develop general categories to indicate the type most relevant to recreational choices.

We created an exposed/sheltered attribute based on the presence of "exposed" or "sheltered" in the ESI "shoretypes" descriptions for any of the landward and two seaward shoreline types. NOAA's type 1 or 2 shoretypes were all classified as exposed; shoretypes 8, 9, or 10 were classified as sheltered. We created variables to indicate whether the shoreline segment had docks or rocks based on the ESI "general line type." We then merged the segments based on these simplified NEWQ attributes, combining like-type adjacent shorelines, resulting in 17,665 segments from Connecticut to Maine (see Figures 1 and 2). We call the resultant shoreline segments "NEWQ shorelines."

The majority of coastal recreation in New England occurs in designated recreation areas, such as major beaches. The U.S. Environmental Protection Agency's (EPA) Beach Program provides grants to assist local authorities in monitoring their coastal beaches and issuing notifications to protect the public from unsafe conditions. The program houses the Beach Advisory and Closing Online Notification (BEACON) system, which provides a database of beach closures and conditions as reported by states. As part of that database, the locations of monitored beaches are provided in the Reach Address Database (RAD), including lines representing the beach area (U.S. EPA, 2021). We first cleaned up and combined the RAD beach lines where they overlapped, then joined them spatially to the NEWQ shorelines. Lastly, we dissolved the NEWQ shorelines where there were RAD beaches occuring across multiple NEWQ shorelines, to maintain beaches as single units. We refer to this resultant set of 17,654 shoreline segments as the "NEWQ shoreline segments." For each of these segments, we summarized the ESI shoreline attributes by the percent of the segment made up of each shoreline type. We then proceeded to summarize attributes of the nearby water and landscape and connect these to the shoreline segments.

THE WATER

The nearshore water in New England varies widely, including open ocean, undeveloped and developed estuaries, urban harbors, and everything in between. This variety offers conditions for a range of recreational activities. Water quality, the primary interest of this study related to recreational activities, also varies with different landward inputs and amounts of ocean tidal influence. The New England states' 303(d) impaired listed waters under the Clean Water Act divides areas of coastal water into management units that reflect geography and hydrology as well as policy and human influence, such as wastewater treatment outflows. Rhode Island, Massachusetts, and New Hampshire use the same divisions for their

shellfish advisory and closure programs. Connecticut uses different divisions for their shellfish program and their 303(d) program. Connecticut's segmentation for their shellfish program matched the other states' 303(d) divisions better than their 303(d) impairment units. Therefore, we used Connecticut's shellfish management coastal water segmentation with the other states' 303(d) listing segments (see Table 2 for dataset references). Maine uses a system that is not comparable to the other New England coastal states, so we did not include Maine in the 303(d) water data for our study.

We merged the New England states' coastal units into a comprehensive set for New England waters, which we refer to as "NEWQ water segments," resulting in 1,732 segments (see Figure 3). We then connected these water segments and their 303(d) impairment status and shellfishing closure status to their adjacent shoreline segments.

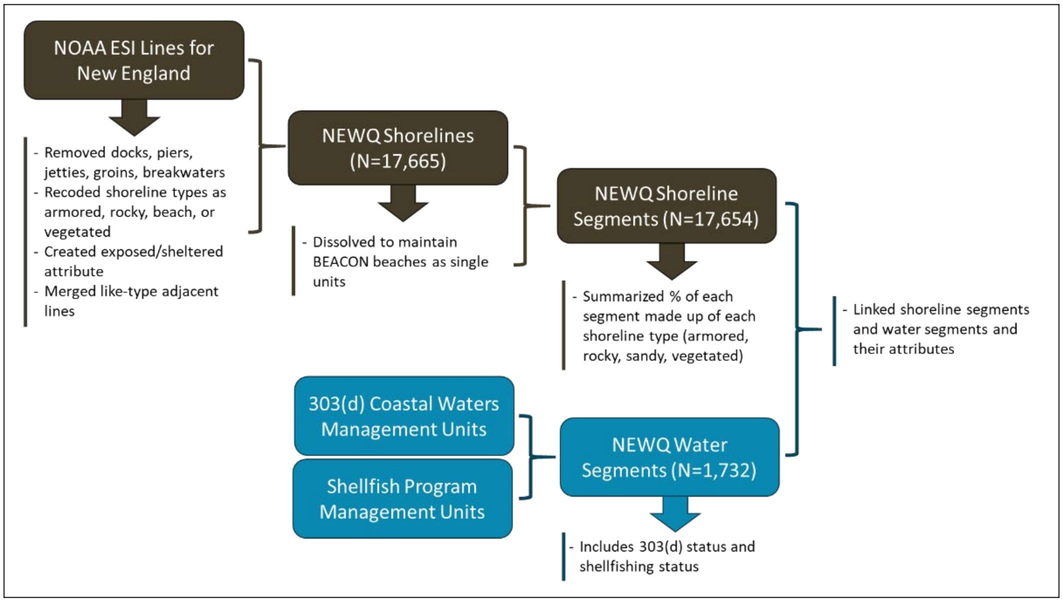

Figure 1. The process of delineating shoreline segments and water segments to compile and match data on shoreline and water attributes.

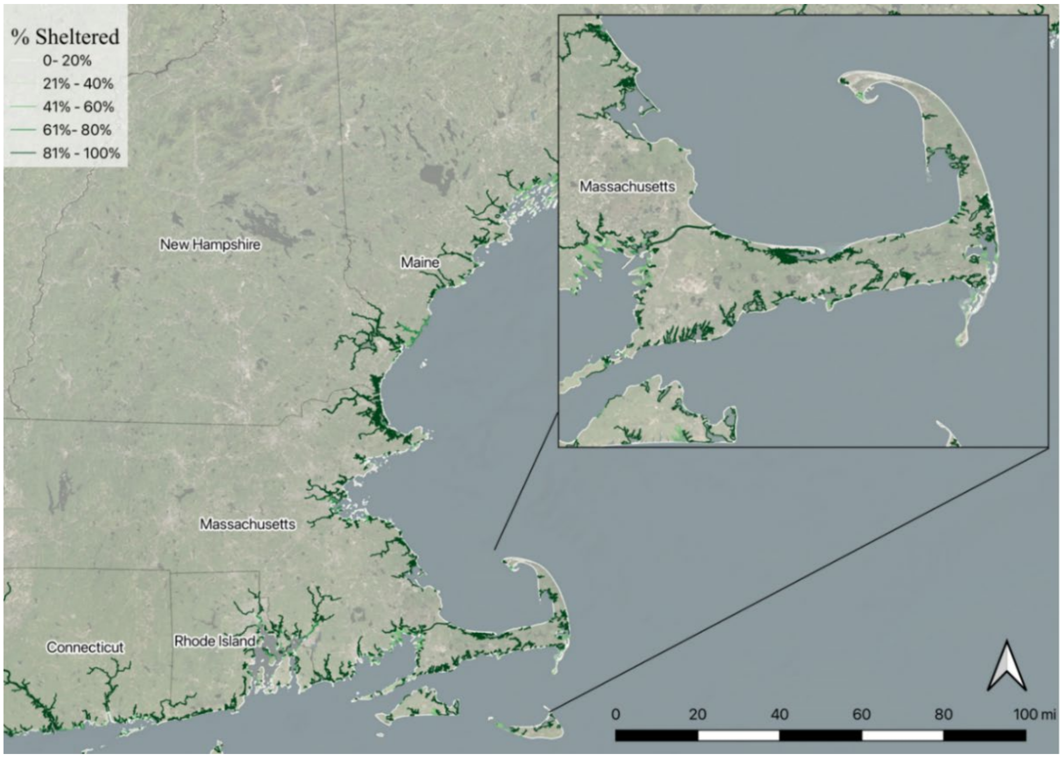

Figure 2. NEWQ sheltered shorelines.

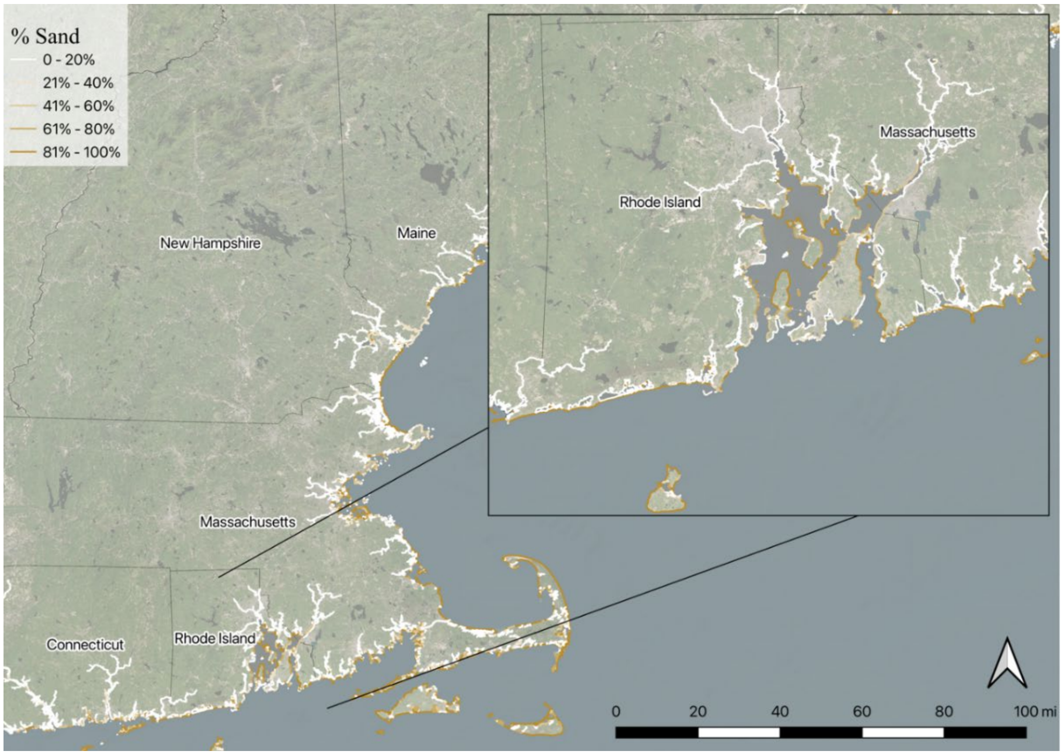

Figure 3. NEWQ sandy shorelines.

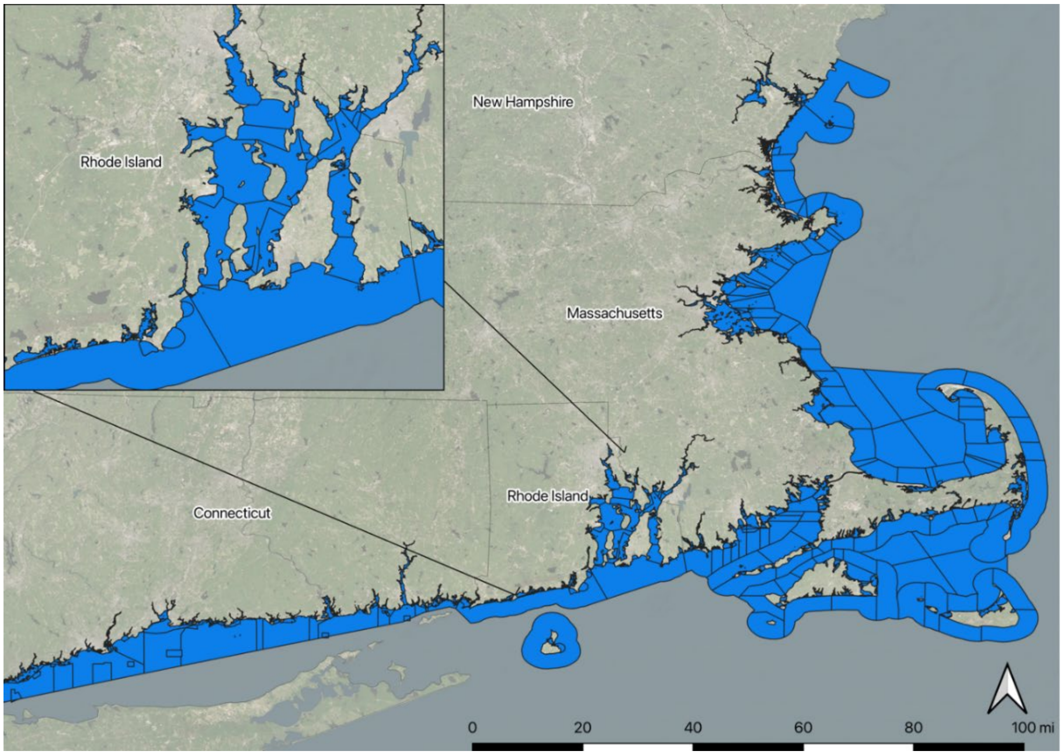

Figure 4. NEWQ water segments.

Table 1. ESI to NEWQ Shoreline Type Crosswalk

Generalized ESI Type	NEWQ shoreline type*
1: Armored	1
1: Armored/2: Rocky and Steep Shorelines (Bedrock/Sand/Clay)	1
1: Armored/2: Rocky and Steep Shorelines (Bedrock/Sand/Clay)/3: Beaches (Sand/Gravel)	3
1: Armored/2: Rocky and Steep Shorelines (Bedrock/Sand/Clay)/4: Flats (Mud/Sand)	1
1: Armored/3: Beaches (Sand/Gravel)	3
1: Armored/3: Beaches (Sand/Gravel)/2: Rocky and Steep Shorelines (Bedrock/Sand/Clay)	3
1: Armored/3: Beaches (Sand/Gravel)/4: Flats (Mud/Sand)	3

Generalized ESI Type	NEWQ shoreline type*
1: Armored/4: Flats (Mud/Sand)	1
1: Armored/5: Vegetated (Grass/Marsh/Mangroves/Scrub-Shrub)	1
1: Armored/5: Vegetated (Grass/Marsh/Mangroves/Scrub-Shrub)/3: Beaches (Sand/Gravel)	1
1: Armored/5: Vegetated (Grass/Marsh/Mangroves/Scrub-Shrub)/4: Flats (Mud/Sand)	1
2: Rocky and Steep Shorelines (Bedrock/Sand/Clay)	2
2: Rocky and Steep Shorelines (Bedrock/Sand/Clay)/3: Beaches (Sand/Gravel)	3
2: Rocky and Steep Shorelines (Bedrock/Sand/Clay)/3: Beaches (Sand/Gravel)/4: Flats (Mud/Sand)	3
2: Rocky and Steep Shorelines (Bedrock/Sand/Clay)/4: Flats (Mud/Sand)	2
2: Rocky and Steep Shorelines (Bedrock/Sand/Clay)/5: Vegetated (Grass/Marsh/Mangroves/Scrub-Shrub)	2
3: Beaches (Sand/Gravel)	3
3: Beaches (Sand/Gravel)/1: Armored	3
3: Beaches (Sand/Gravel)/1: Armored/4: Flats (Mud/Sand)	3
3: Beaches (Sand/Gravel)/2: Rocky and Steep Shorelines (Bedrock/Sand/Clay)	3
3: Beaches (Sand/Gravel)/2: Rocky and Steep Shorelines (Bedrock/Sand/Clay)/4: Flats (Mud/Sand)	3
3: Beaches (Sand/Gravel)/4: Flats (Mud/Sand)	3
3: Beaches (Sand/Gravel)/5: Vegetated (Grass/Marsh/Mangroves/Scrub-Shrub)	3
3: Beaches (Sand/Gravel)/5: Vegetated (Grass/Marsh/Mangroves/Scrub-Shrub)/4: Flats (Mud/Sand)	3
5: Vegetated (Grass/Marsh/Mangroves/Scrub-Shrub)	5
5: Vegetated (Grass/Marsh/Mangroves/Scrub-Shrub)/1: Armored	5
5: Vegetated (Grass/Marsh/Mangroves/Scrub-Shrub)/1: Armored/3: Beaches (Sand/Gravel)	5

Generalized ESI Type	NEWQ shoreline type*
5: Vegetated (Grass/Marsh/Mangroves/Scrub-Shrub)/1: Armored/4: Flats (Mud/Sand)	5
5: Vegetated (Grass/Marsh/Mangroves/Scrub-Shrub)/2: Rocky and Steep Shorelines (Bedrock/Sand/Clay)	5
5: Vegetated (Grass/Marsh/Mangroves/Scrub-Shrub)/2: Rocky and Steep Shorelines (Bedrock/Sand/Clay)/3: Beaches (Sand/Gravel)	5
5: Vegetated (Grass/Marsh/Mangroves/Scrub-Shrub)/2: Rocky and Steep Shorelines (Bedrock/Sand/Clay)/4: Flats (Mud/Sand)	5
5: Vegetated (Grass/Marsh/Mangroves/Scrub-Shrub)/3: Beaches (Sand/Gravel)	3
5: Vegetated (Grass/Marsh/Mangroves/Scrub-Shrub)/3: Beaches (Sand/Gravel)/2: Rocky and Steep Shorelines (Bedrock/Sand/Clay)	5
5: Vegetated (Grass/Marsh/Mangroves/Scrub-Shrub)/3: Beaches (Sand/Gravel)/4: Flats (Mud/Sand)	5
5: Vegetated (Grass/Marsh/Mangroves/Scrub-Shrub)/4: Flats (Mud/Sand)	5

*NEWQ Types: 1 = armored; 2=rocky/steep; 3=beach; 4=flats; 5=vegetated

Table 2. Input Datasets

Attribute		Description	Source	References
Shoreline				
	Types	Sandy, vegetated, rocky, armored. exposed/sheltered	NOAA ESI	https://response.restoration.noaa.gov/resources/environmental-sensitivity-index-esi-maps
	Beaches	Beach locations	EPA BEACON	https://www.epa.gov/waterdata/beacon-20-beach-advisory-and-closing-online-notification
Water				
	303d Impairments	Impairments to coastal waters from 303d listings	State GIS portals	RI: 2010- https://www.rigis.org/datasets/marine-and-estuarine-waters-ri-integrated-water-quality-monitoring-assessment-2010/explore MA: 2014- https://docs.digital.mass.gov/dataset/massgis-data-massdep-2014-integrated-list-waters-305b303d NH: 2016- http://nhdes.maps.arcgis.com/apps/webappviewer/index.html?id=aca7a13dced5426aa542c62b1ea10d0c CT:2016- https://portal.ct.gov/DEEP/GIS-and-Maps/Data/GIS-DATA#NaturalResourcesManagement

Qualitative Coding of Open-Ended Responses

Table 3 lists the qualitative coding of responses to an open-ended question on the New England Coastal Recreation Survey "Why did you choose that place?" In their responses, survey participants identified between zero and five reasons for why they chose the particular location for their visit. The responses were qualitatively coded by one researcher in MS Excel, using codes as shown in Table 3.

Table 3. Coding for Q2_4_A and Q2_16_B: Why did you choose that place?

**Qualitative Code**	**Qualitative Code Description**
Access	Access, easy driving, other beach full, public landing, public transportation
Activity	Allowed for the desired activity, boat moored
Affordable	Affordable, cost-free
Built features	Pools, lighthouses, food, historical features, parking, towns
Clean	Neat, clean, well kept
Close	Close, local, I live here
Convenience	Convenience, location
Educational and Conservation	Noted as conservation areas or for educational programs
Event	Marathon, wedding
Family	visiting family, family has pass/home, kid friendly, family members identified
Familiarity	Hometown
Friends	friends specifically identified
Leisure	Vacation, fun
Less crowded	Fewer people, quiet, not crowded
Natural features	Specific features of the environment identified, ex beach and waves, scenery
New	See a new place
People	(as a positive) Not identified as friends or family
Pets	Dogs allowed

Qualitative Code	Qualitative Code Description
Popular	Popular
Random	Random, Googled, don't know, rental company, someone else recommended it
Safety	Safe, away from street
Season tickets	Passes, member, resident beach
Spiritual and Personal	Memories, favorite, refreshing, peaceful, beautiful, fun, interesting

Survey Data and Coding

The data files and code presented in this study can be found in a zenodo.org repository at: https://doi.org/10.5281/zenodo.5807859.

REFERENCES

U.S. EPA. 2021. Beach Advisory and Closing Online Notification (BEACON). https://www.epa.gov/waterdata/beacon-20-beach-advisory-and-closing-online-notification#:~:text=The%20U.S.%20Environmental%20Protection%20Agency,occurrences%20for%20coastal%20recreation%20waters. Accessed August 16, 2021

NOAA Office of Response and Restoration. 2019. Environmental Sensitivity Index (ESI) Maps and Data. https://response.restoration.noaa.gov/resources/environmental-sensitivity-index-esi-maps. Accessed August 9, 2021.
